# Supplementary figures and images for: Acetyl-CoA production by specific metabolites promotes cardiac repair after myocardial infarction via histone acetylation
Source: eLife. 2021 Dec 23;10:e60311. doi: 10.7554/eLife.60311 (PMC8763402; doi:10.7554/eLife.60311)

Figure 1-source data 2 Original western blot figures for Figure 1J

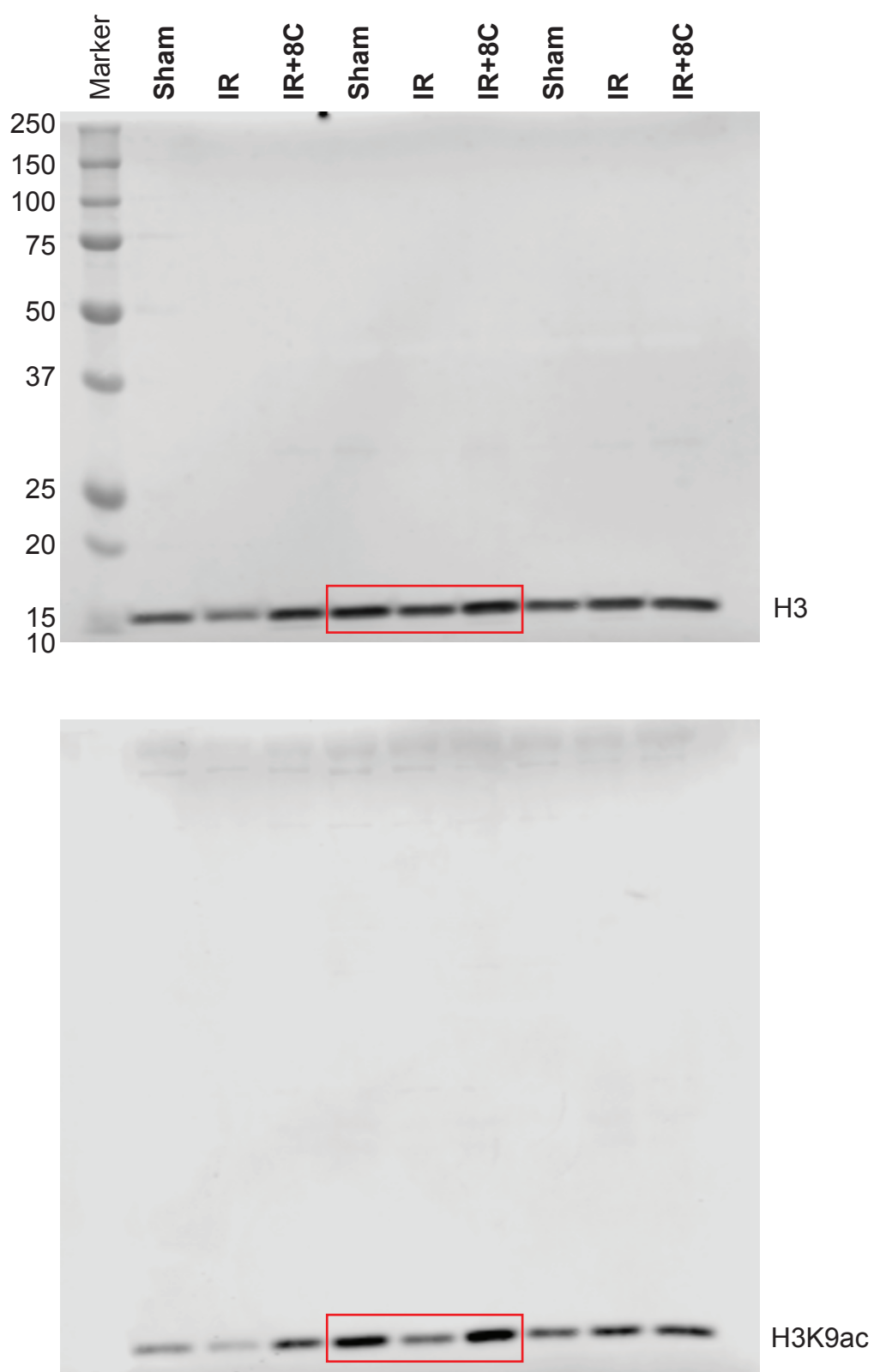

Supplement: Figure 1—source data 2. [file elife-60311-fig1-data2.zip › Figure 1 source data 2/Figure 1-source data 2.pdf]

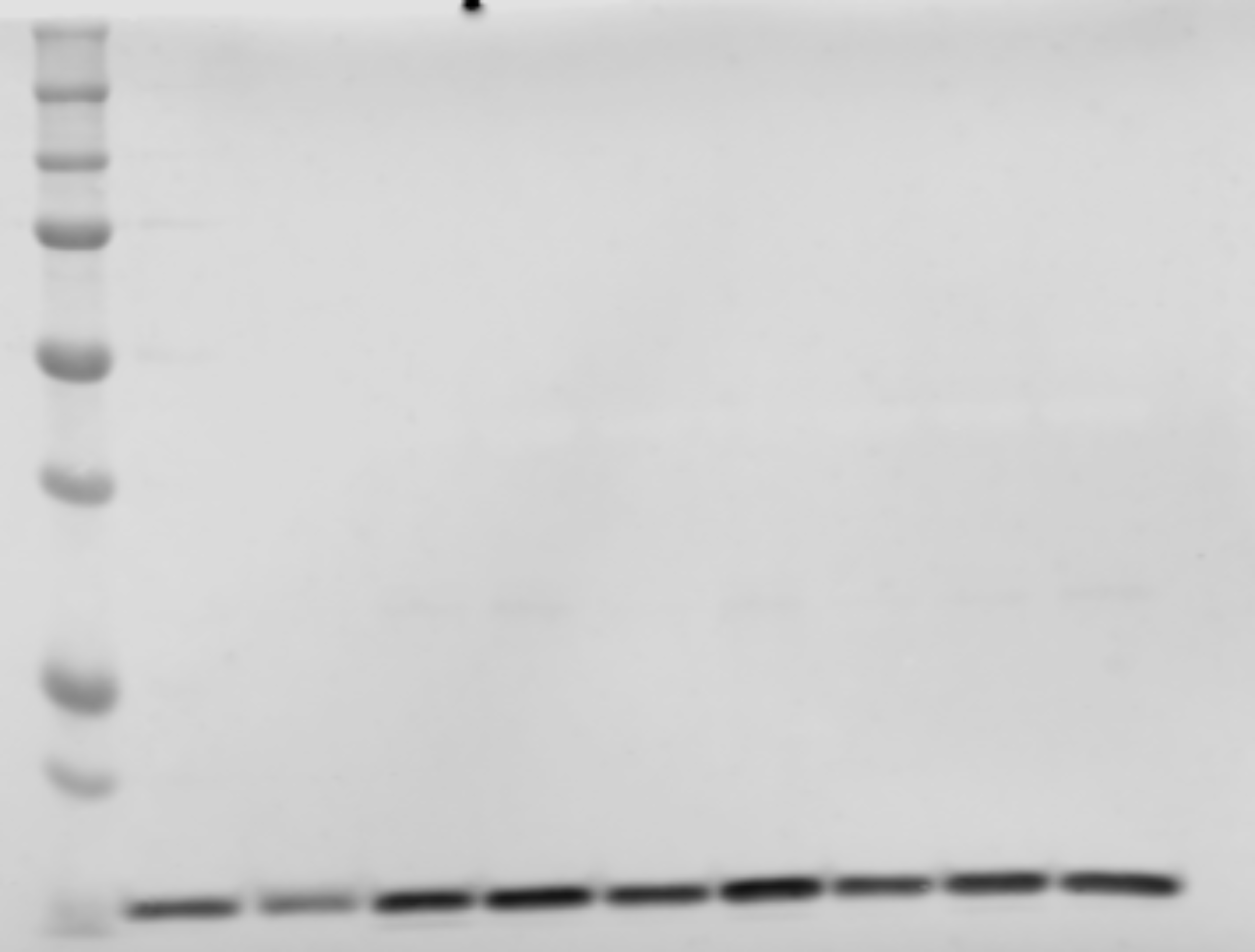

Supplement: Figure 1—source data 2. [file elife-60311-fig1-data2.zip › Figure 1 source data 2/H3.png]

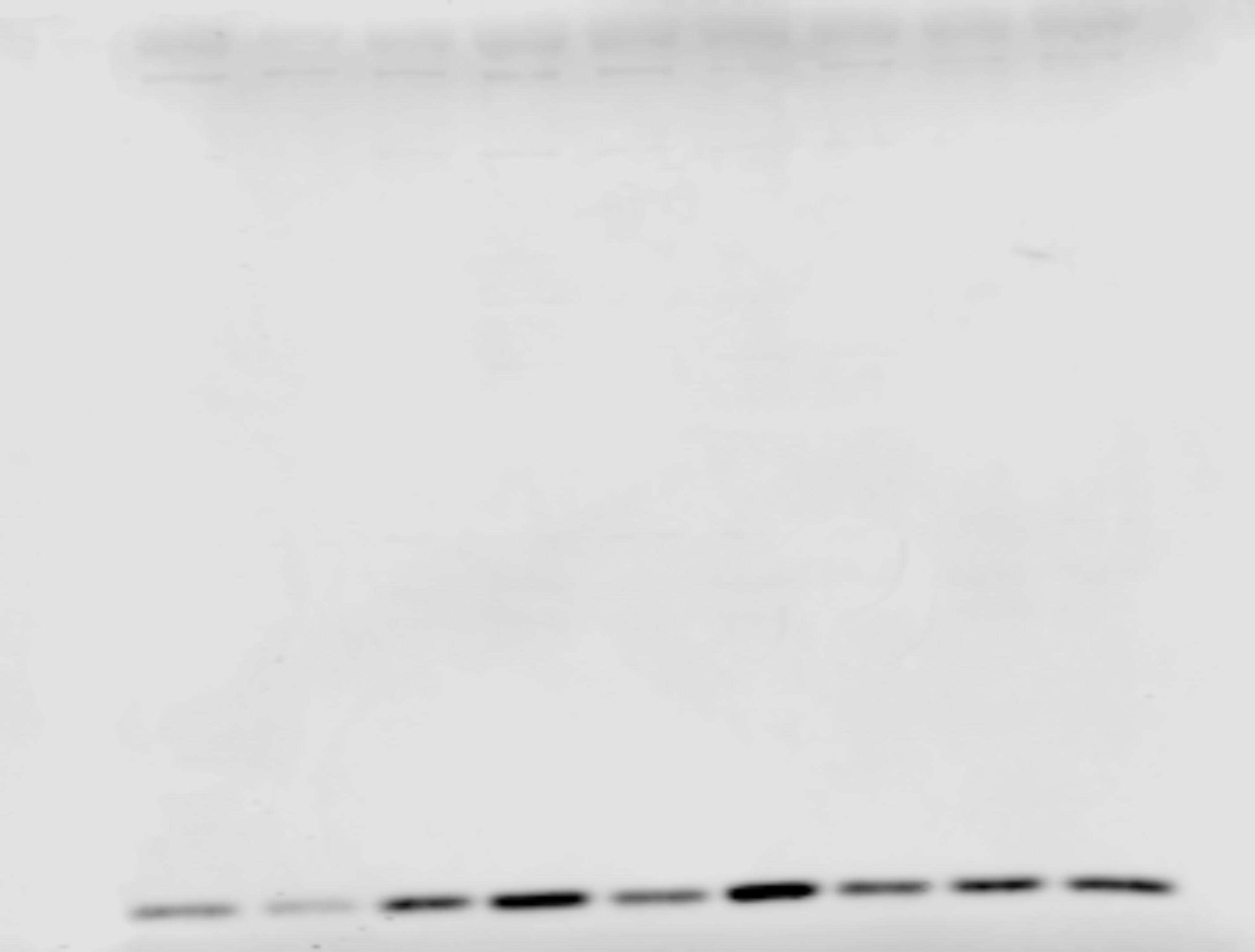

Supplement: Figure 1—source data 2. [file elife-60311-fig1-data2.zip › Figure 1 source data 2/H3K9.png]

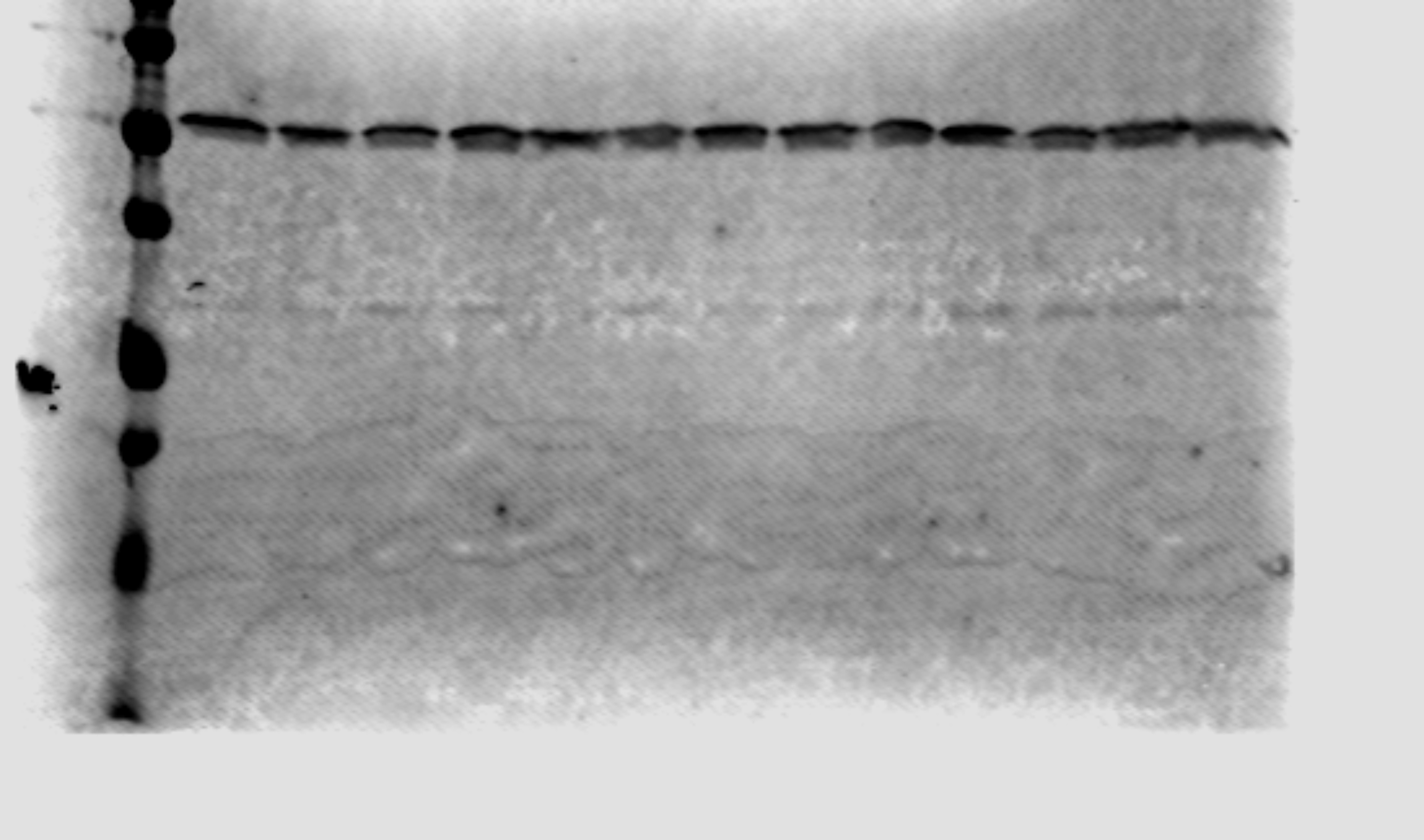

Supplement: Figure 2—source data 2. [file elife-60311-fig2-data2.zip › Figure 2-source data 2/bax_tub.png]

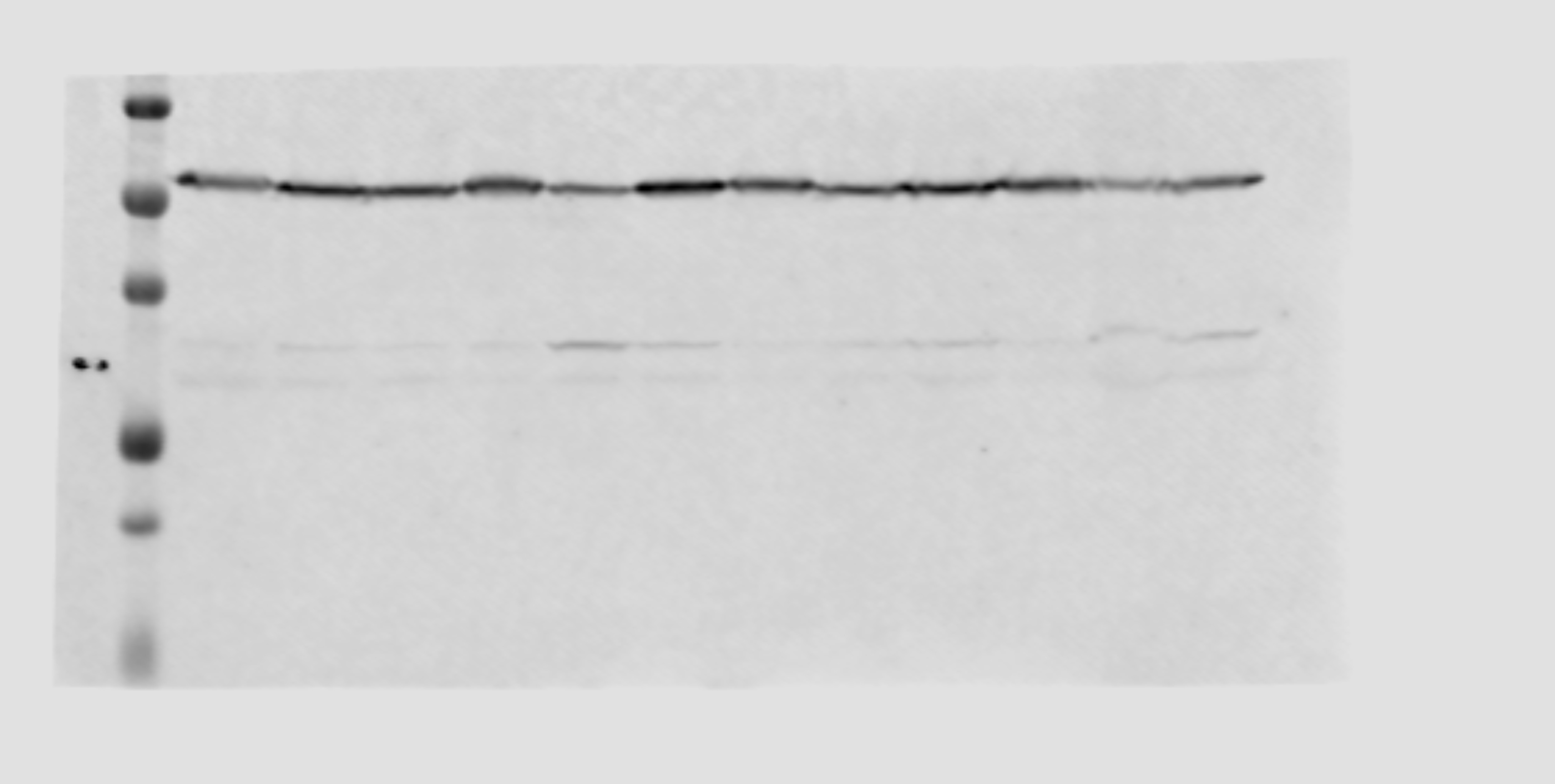

Supplement: Figure 2—source data 2. [file elife-60311-fig2-data2.zip › Figure 2-source data 2/bcl2_tub.png]

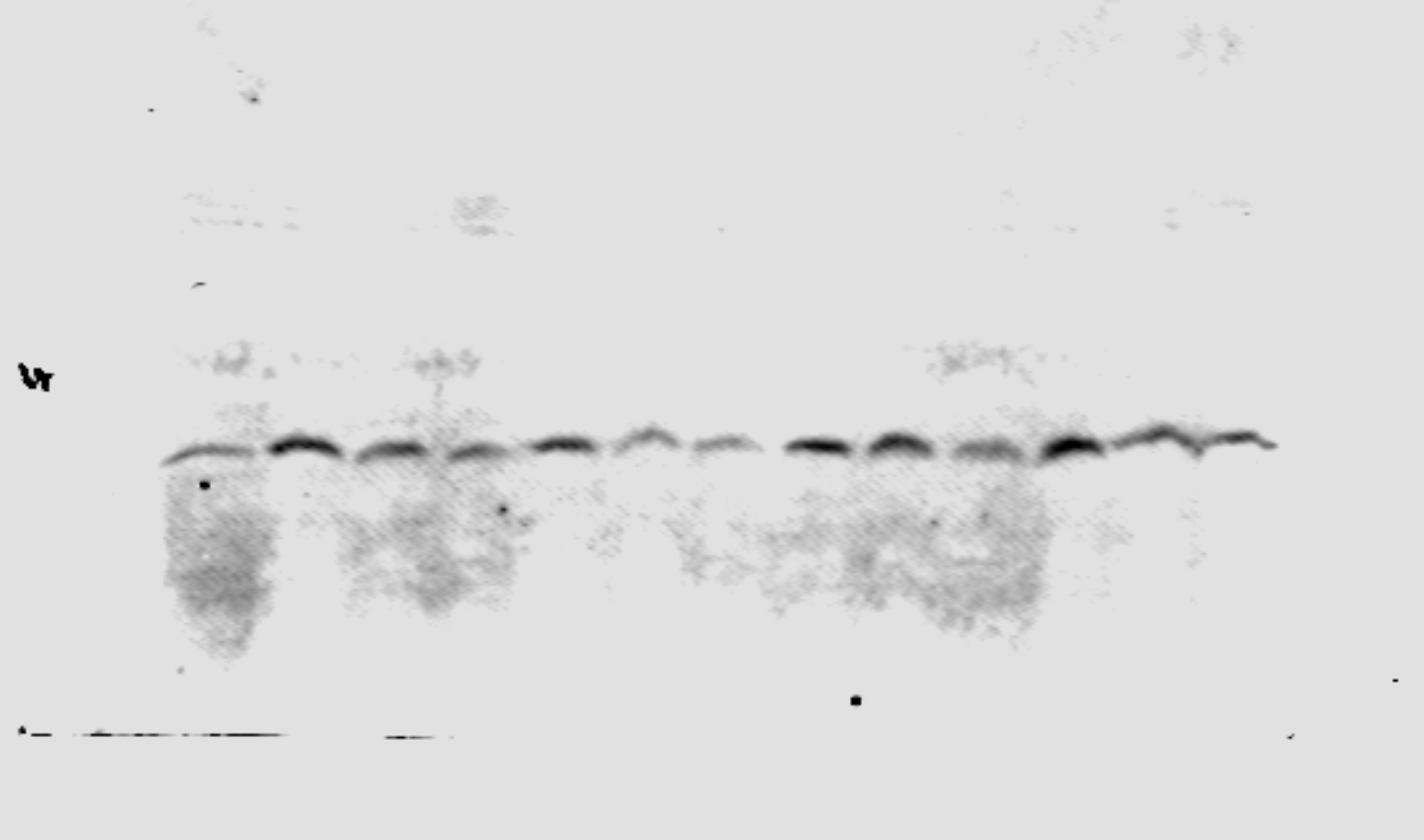

Supplement: Figure 2—source data 2. [file elife-60311-fig2-data2.zip › Figure 2-source data 2/bax.png]

Figure 2-source data 2 Original western blot figures for Figure 2E

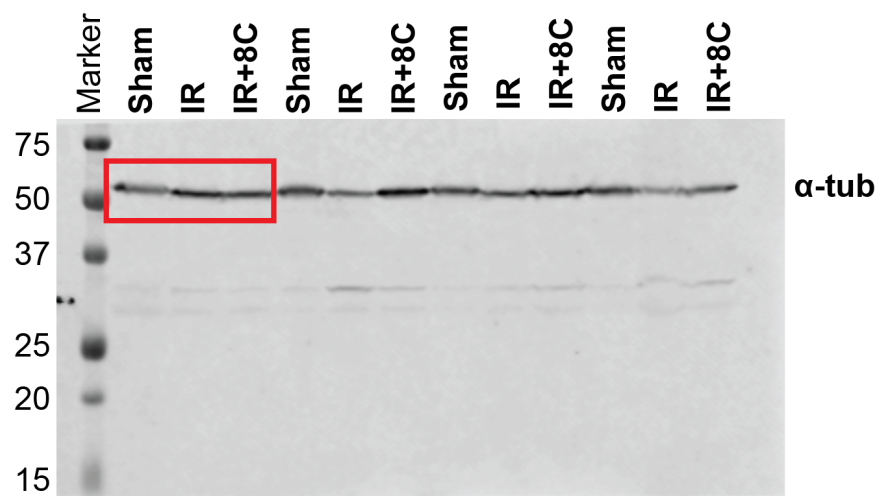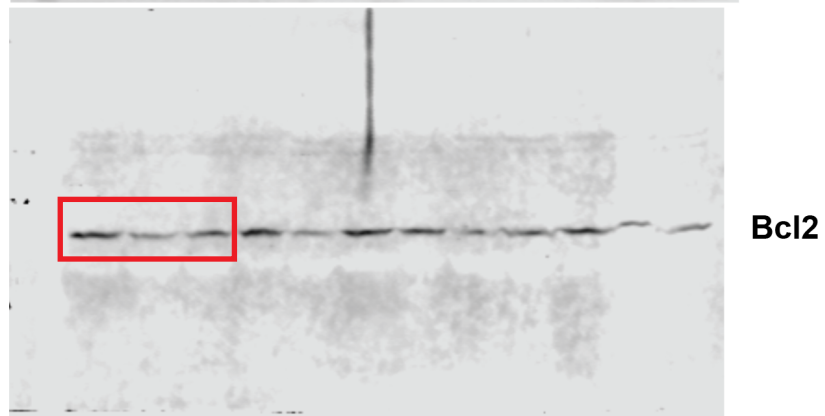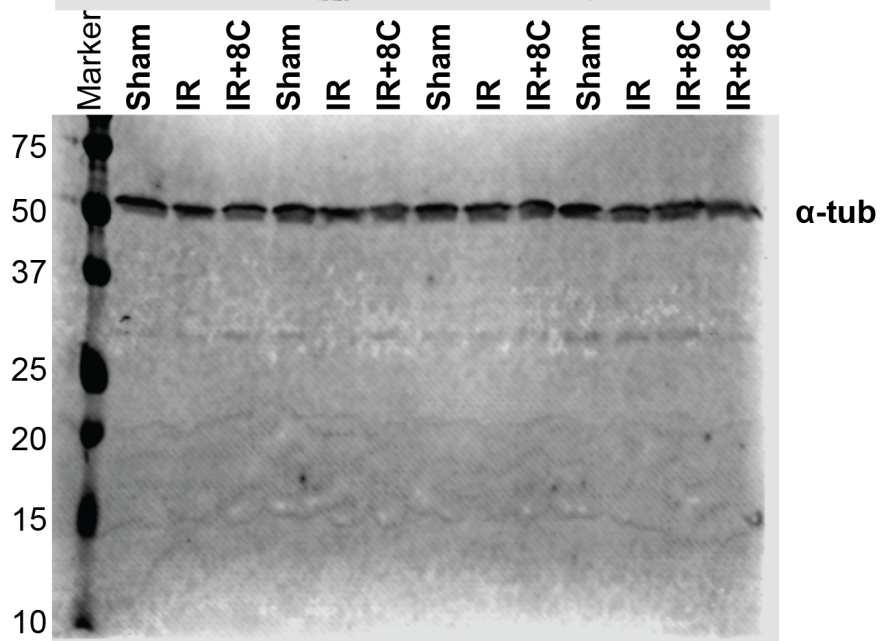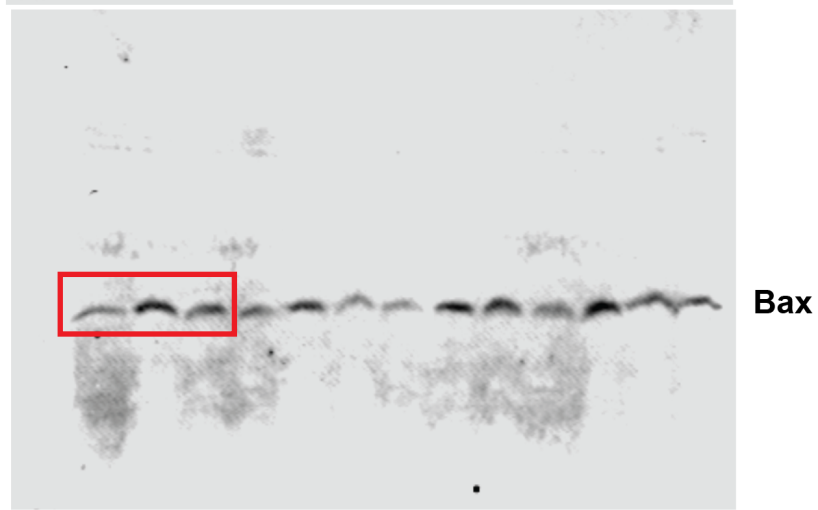

Supplement: Figure 2—source data 2. [file elife-60311-fig2-data2.zip › Figure 2-source data 2/Figure 2-source data 2.pdf]

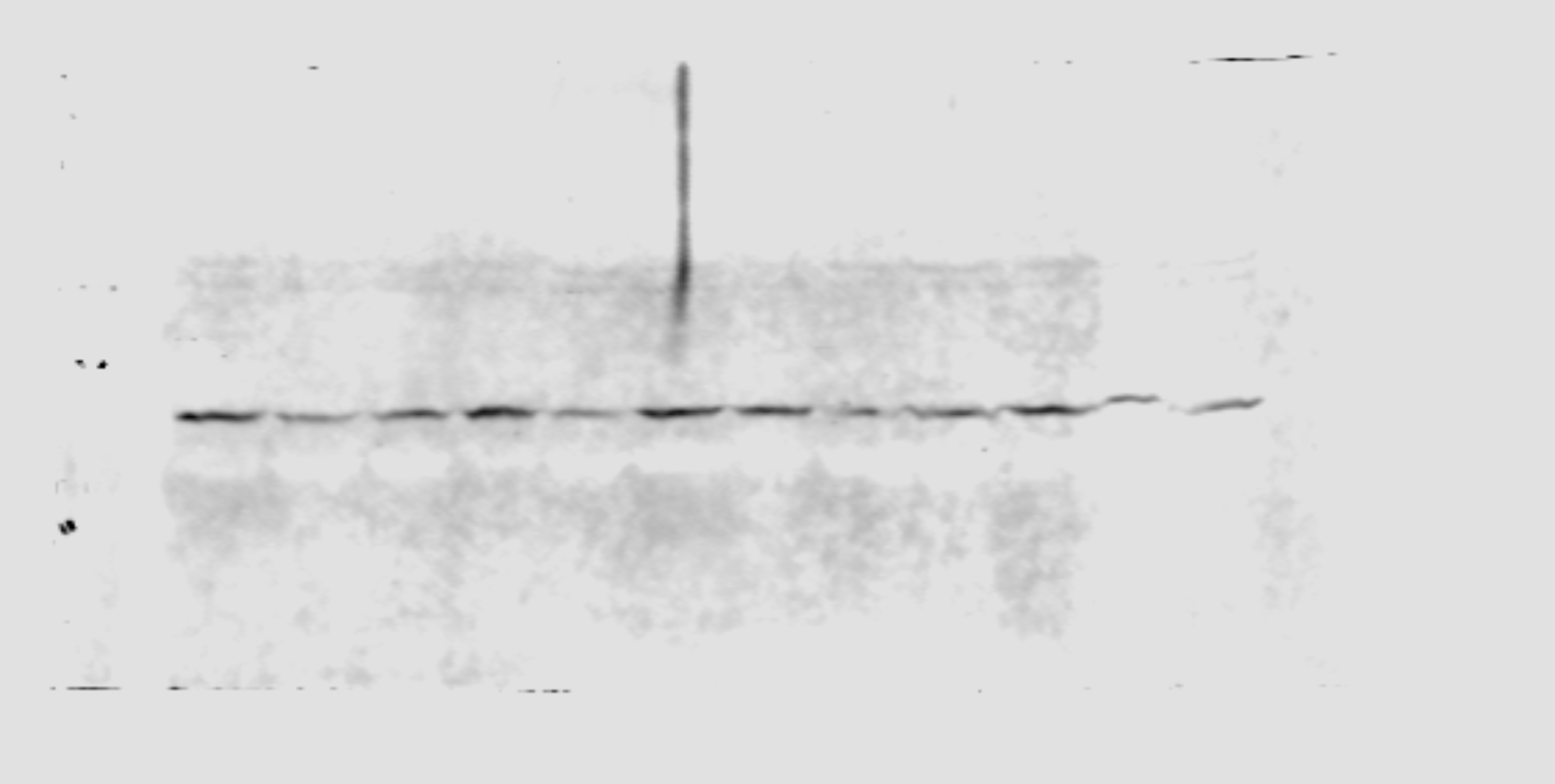

Supplement: Figure 2—source data 2. [file elife-60311-fig2-data2.zip › Figure 2-source data 2/bcl2.png]

Figure 3-source data 2 Original western blot figures for Figure 3E

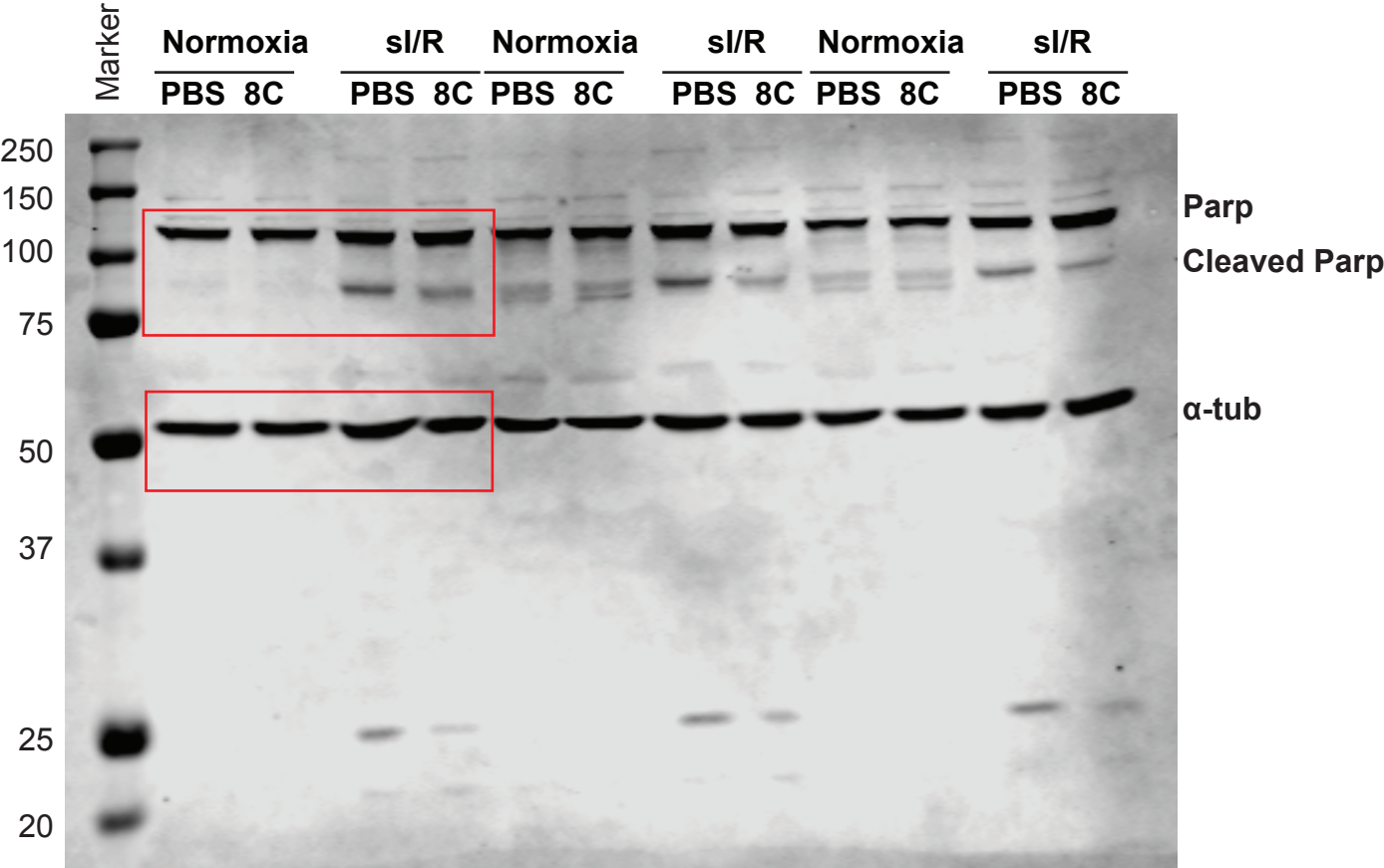

Supplement: Figure 3—source data 2. [file elife-60311-fig3-data2.zip › Figure 3-source data 2/Figure 3-source data 2.pdf]

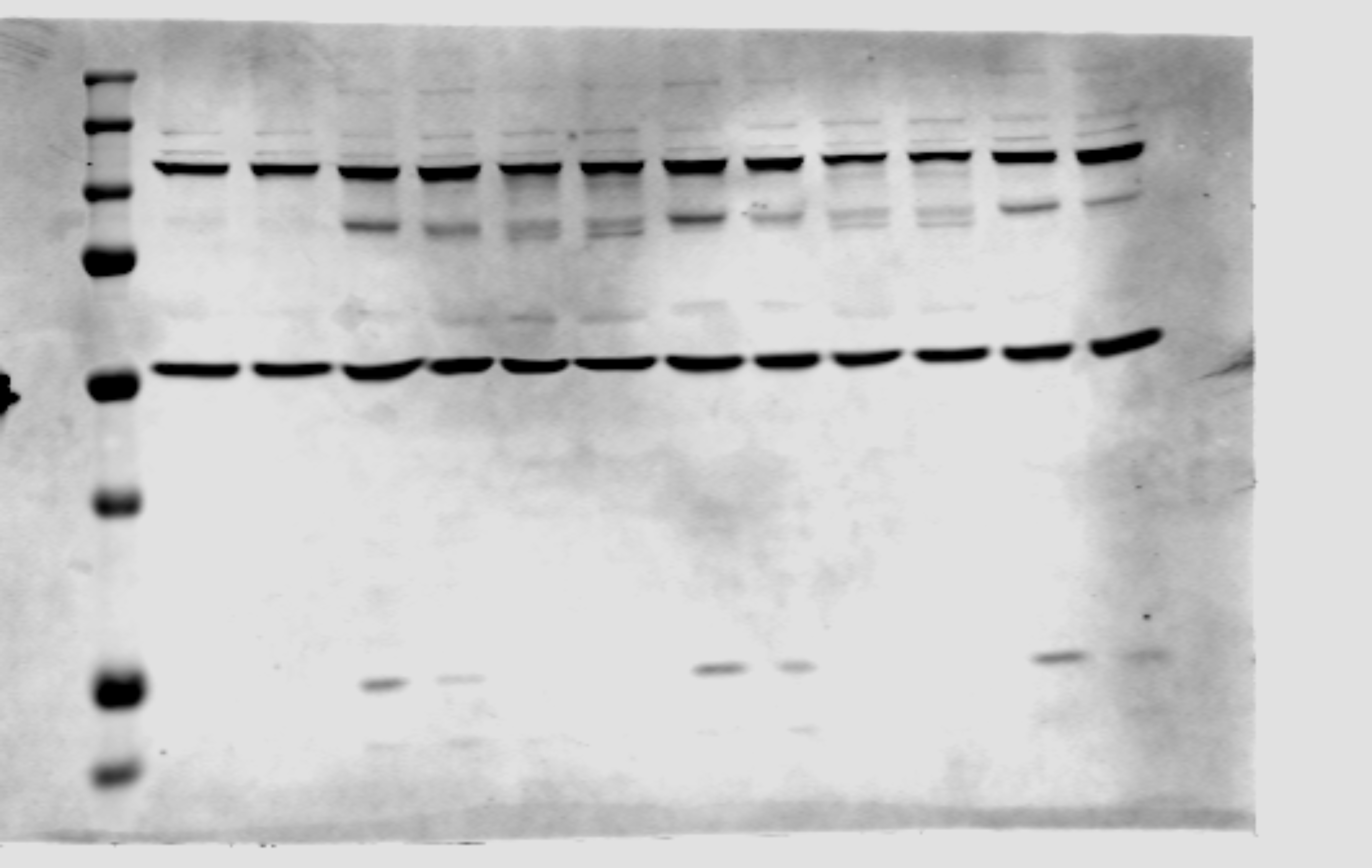

Supplement: Figure 3—source data 2. [file elife-60311-fig3-data2.zip › Figure 3-source data 2/Parp.png]

Figure 4-source data 2 Original western blot for Figure 4

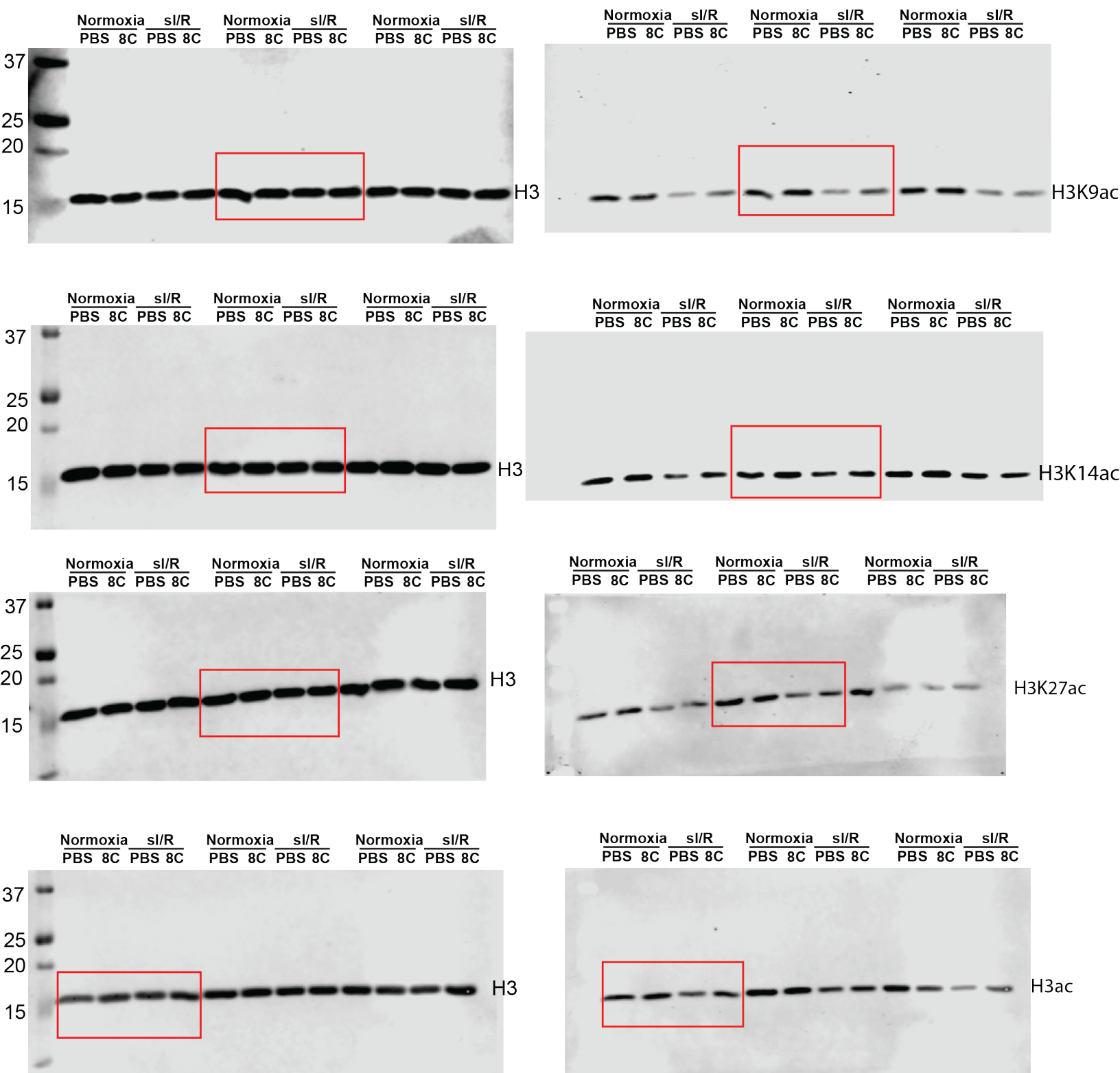

Supplement: Figure 4—source data 2. [file elife-60311-fig4-data2.zip › Figure 4-source data 2/Figure 4-source data 2.pdf]

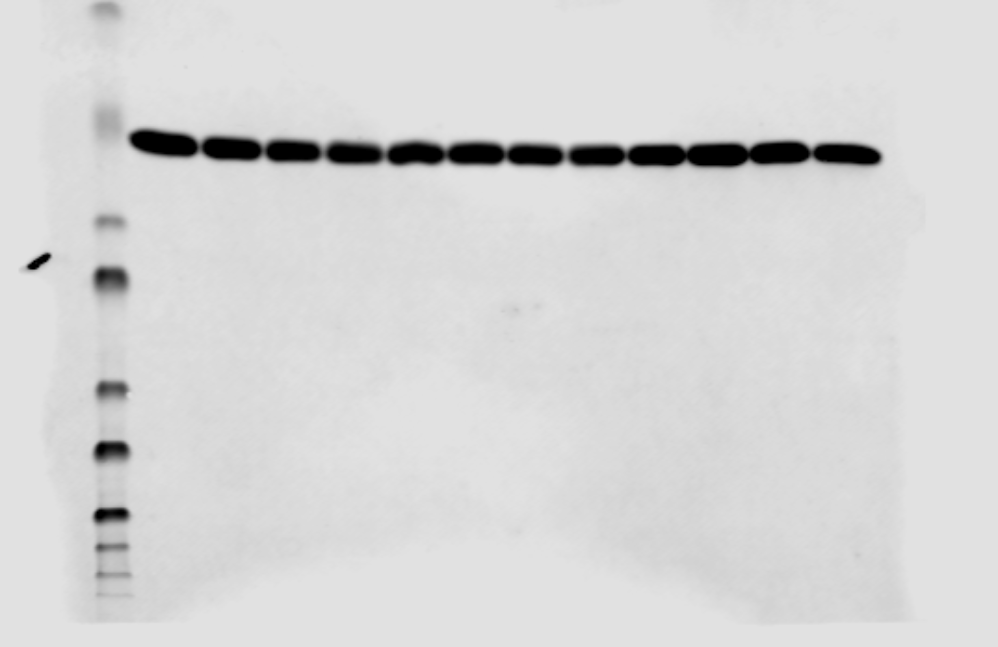

Supplement: Figure 4—source data 2. [file elife-60311-fig4-data2.zip › Figure 4-source data 2/H3K14ac_H3.png]

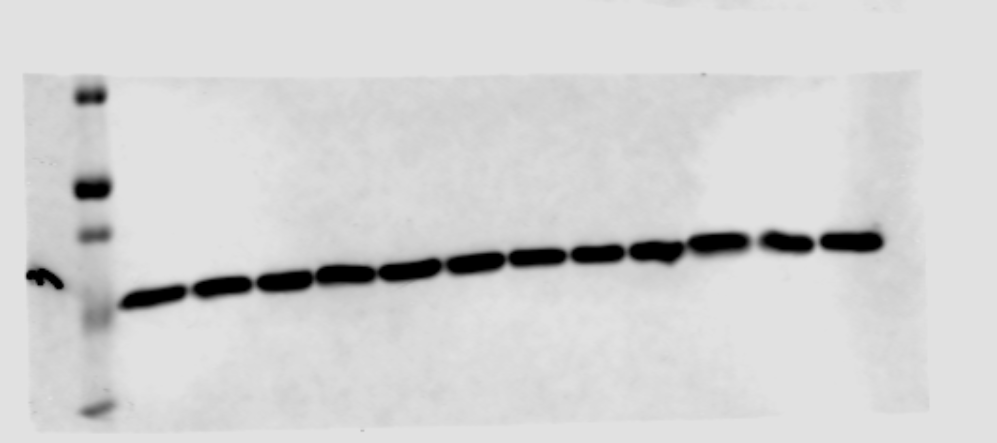

Supplement: Figure 4—source data 2. [file elife-60311-fig4-data2.zip › Figure 4-source data 2/H3K27ac_H3.png]

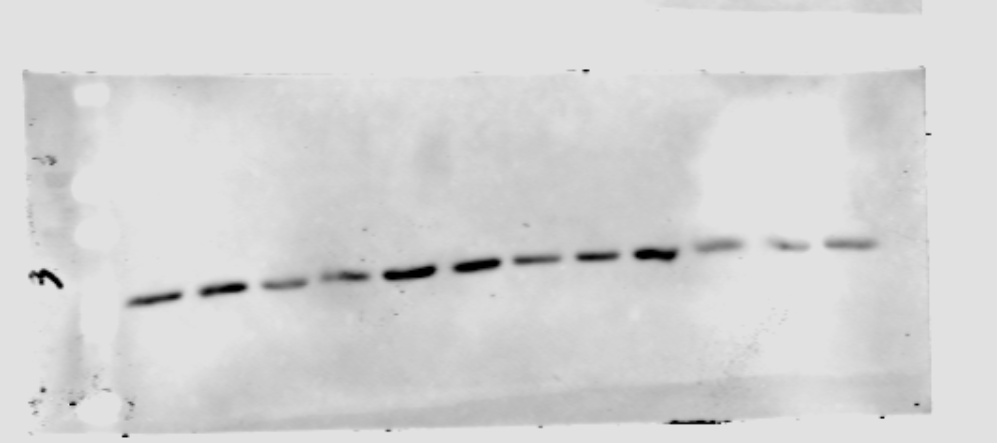

Supplement: Figure 4—source data 2. [file elife-60311-fig4-data2.zip › Figure 4-source data 2/H3K27ac.png]

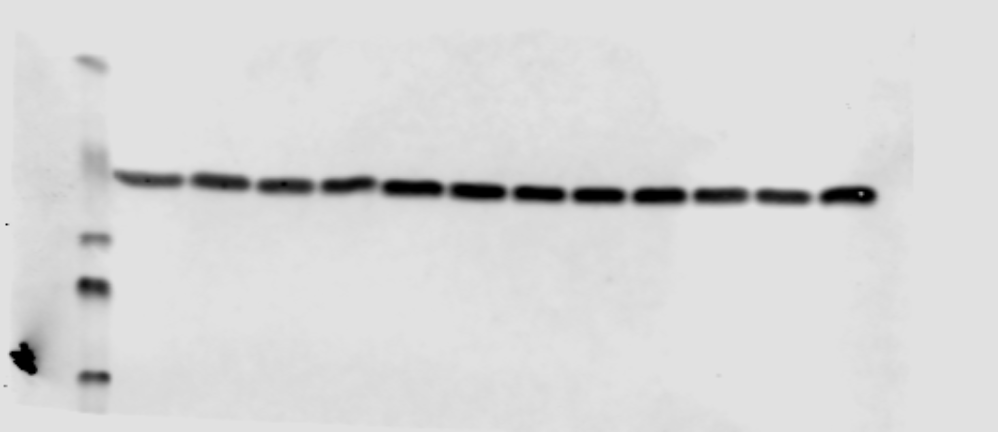

Supplement: Figure 4—source data 2. [file elife-60311-fig4-data2.zip › Figure 4-source data 2/acH3_h3.png]

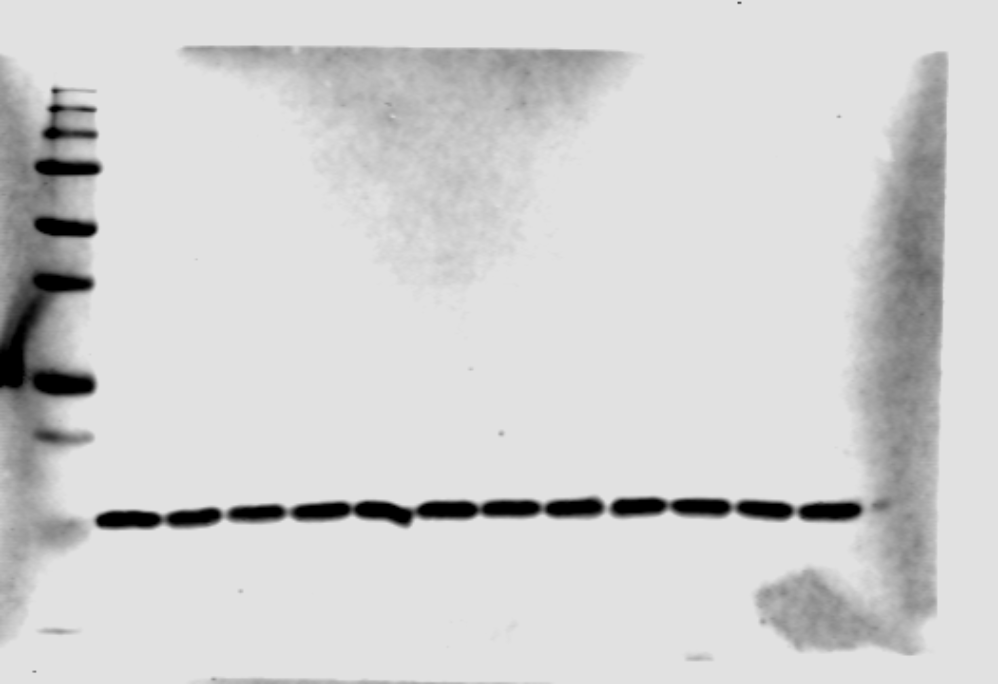

Supplement: Figure 4—source data 2. [file elife-60311-fig4-data2.zip › Figure 4-source data 2/H3K9ac_H3.png]

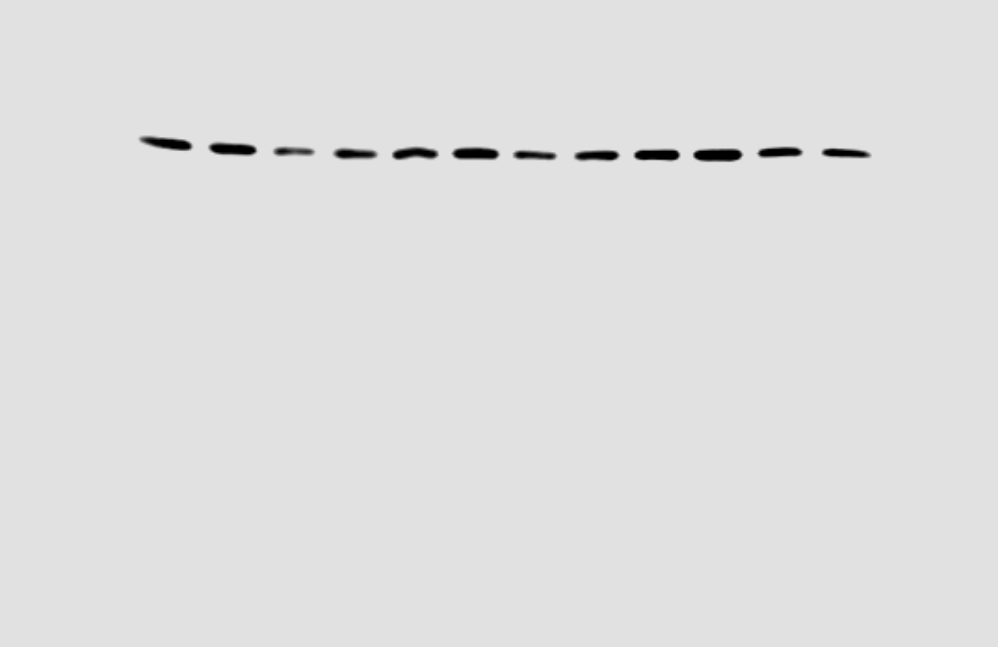

Supplement: Figure 4—source data 2. [file elife-60311-fig4-data2.zip › Figure 4-source data 2/H3K14ac.png]

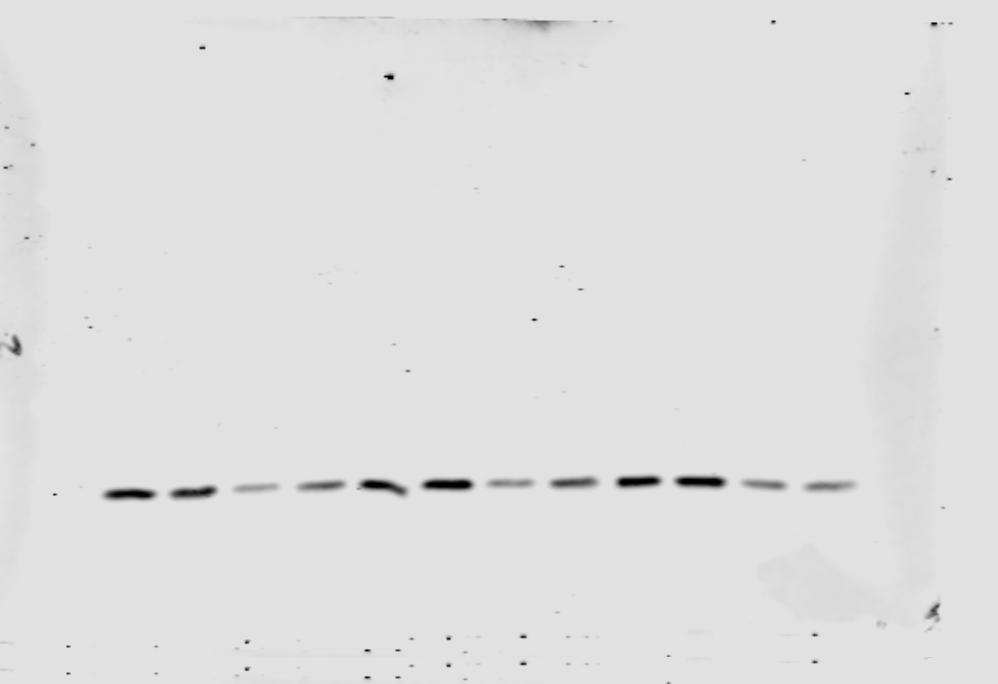

Supplement: Figure 4—source data 2. [file elife-60311-fig4-data2.zip › Figure 4-source data 2/H3K9ac.png]

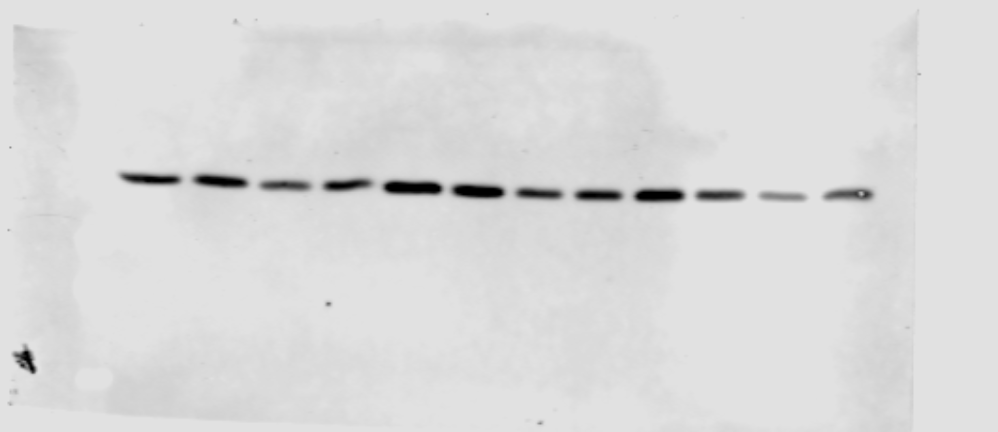

Supplement: Figure 4—source data 2. [file elife-60311-fig4-data2.zip › Figure 4-source data 2/acH3.png]

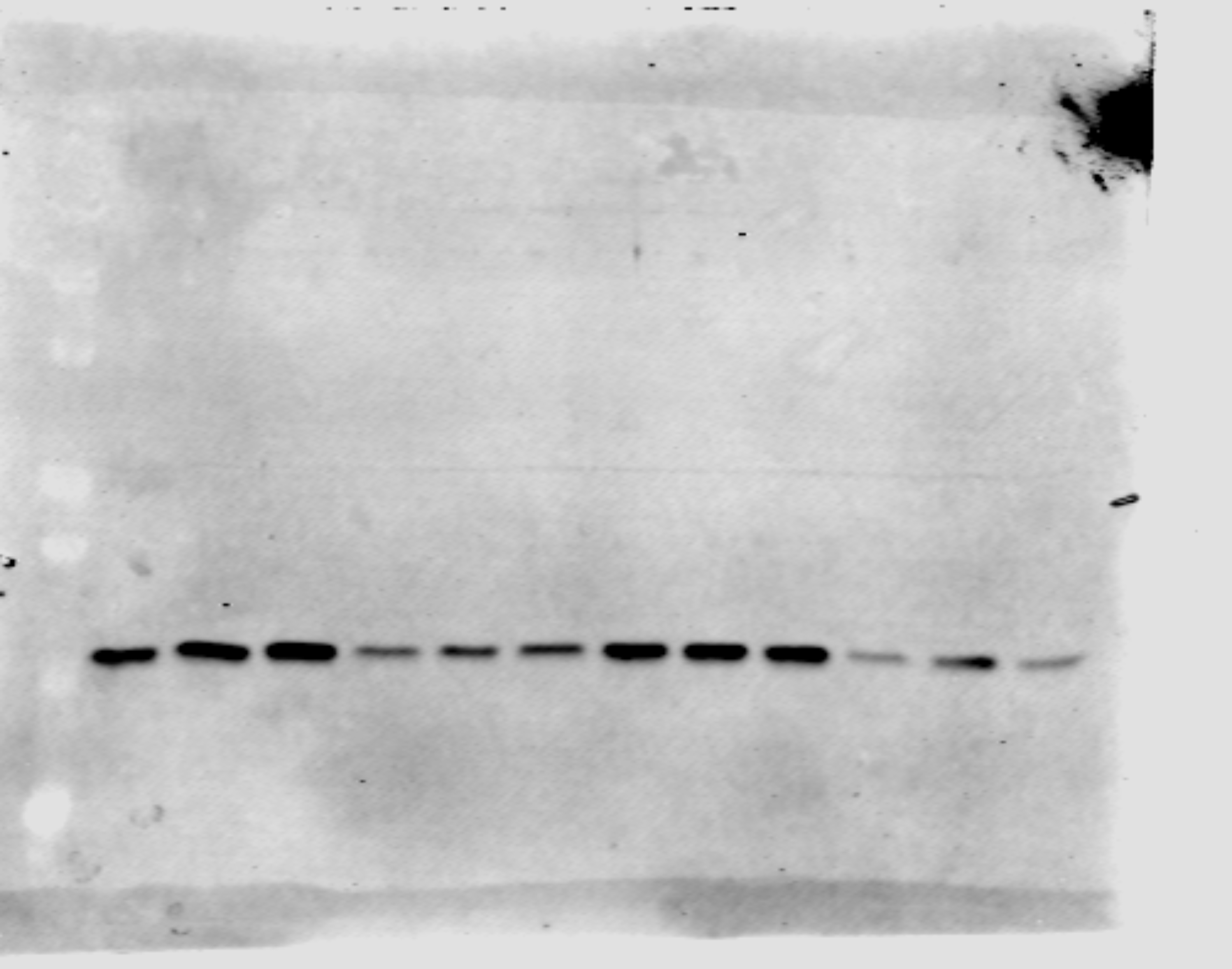

Supplement: Figure 4—figure supplement 1—source data 1. [file elife-60311-fig4-figsupp1-data1.zip › Figure 4-figure supplement 1-source data 1/H3K9_ac.png]

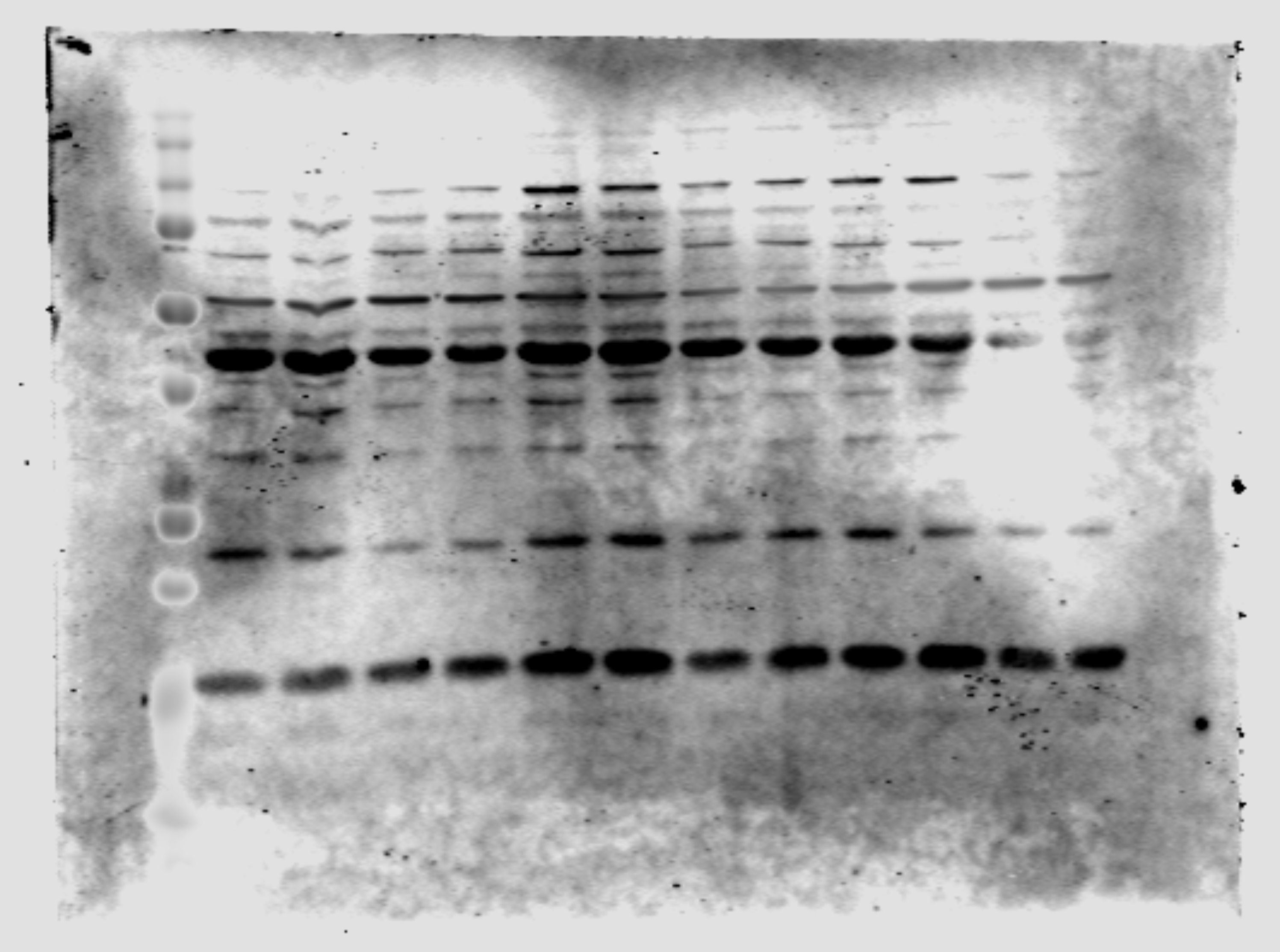

Supplement: Figure 4—figure supplement 1—source data 1. [file elife-60311-fig4-figsupp1-data1.zip › Figure 4-figure supplement 1-source data 1/NQO1.png]

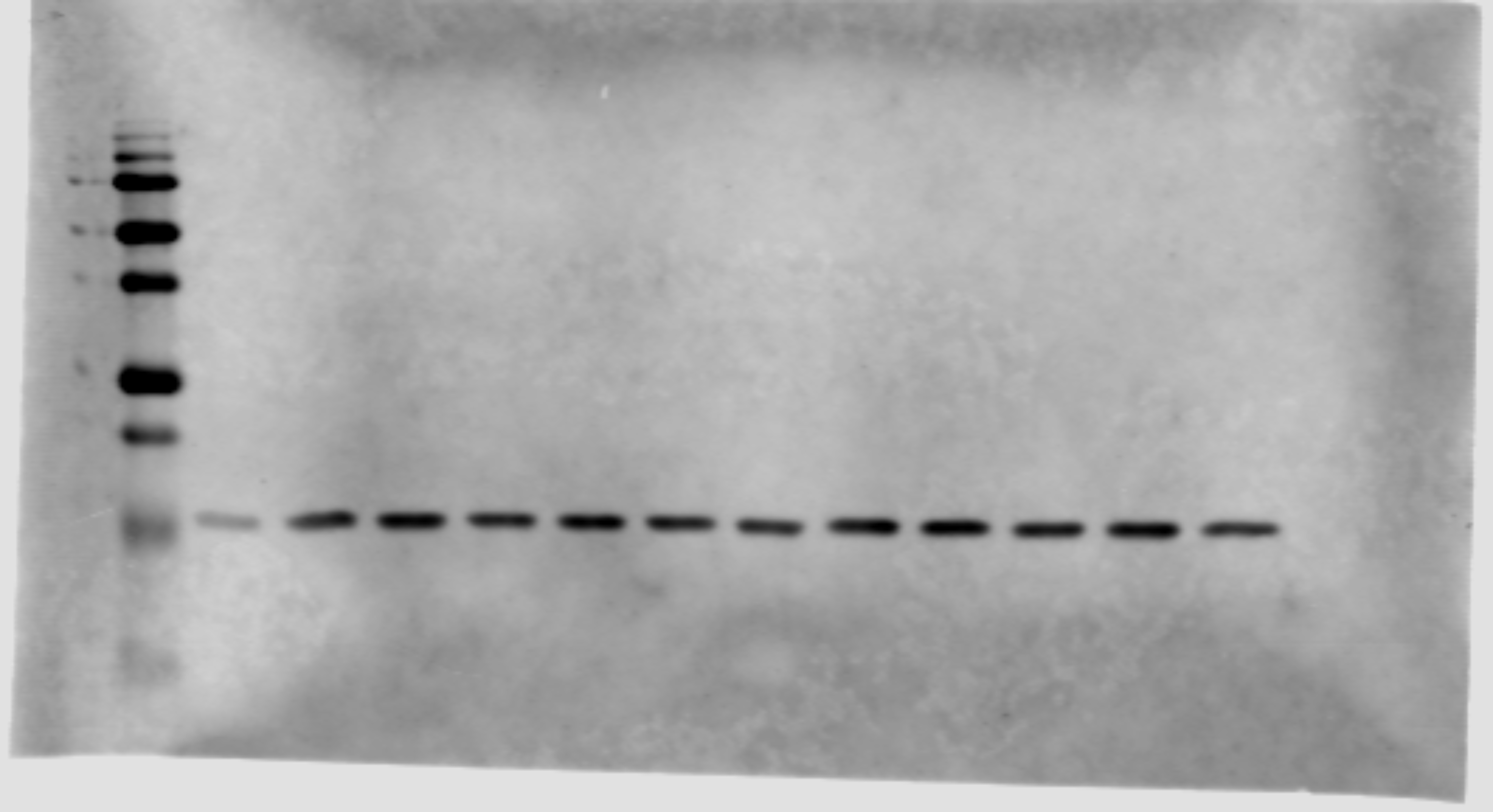

Supplement: Figure 4—figure supplement 1—source data 1. [file elife-60311-fig4-figsupp1-data1.zip › Figure 4-figure supplement 1-source data 1/acH3_H3.png]

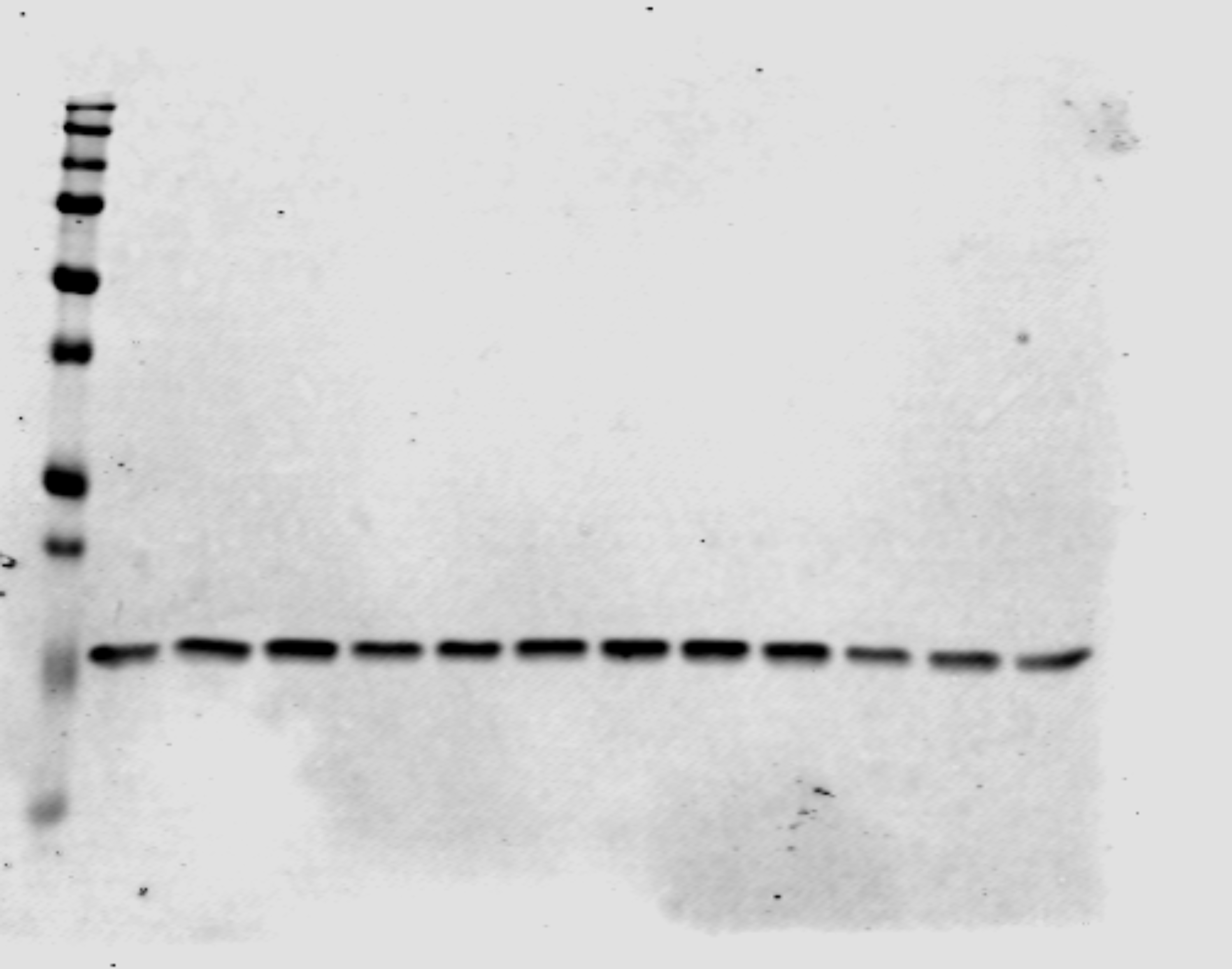

Supplement: Figure 4—figure supplement 1—source data 1. [file elife-60311-fig4-figsupp1-data1.zip › Figure 4-figure supplement 1-source data 1/H3K9ac_H3.png]

Figure 4-figure supplement 1- source data 1: Original blots for Figure 4-figure supplement 1

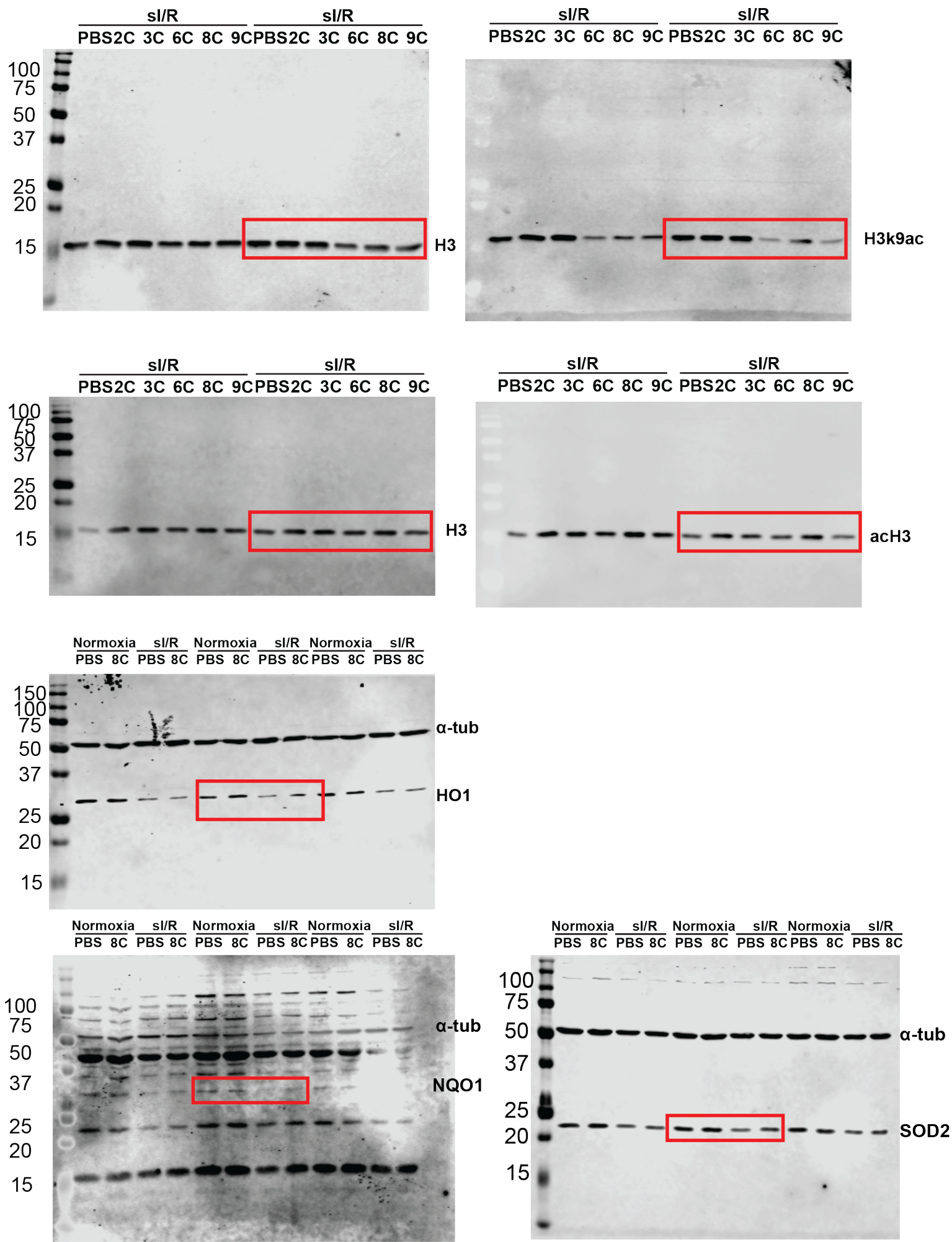

Supplement: Figure 4—figure supplement 1—source data 1. [file elife-60311-fig4-figsupp1-data1.zip › Figure 4-figure supplement 1-source data 1/Figure 4-figures supplement source data 1.pdf]

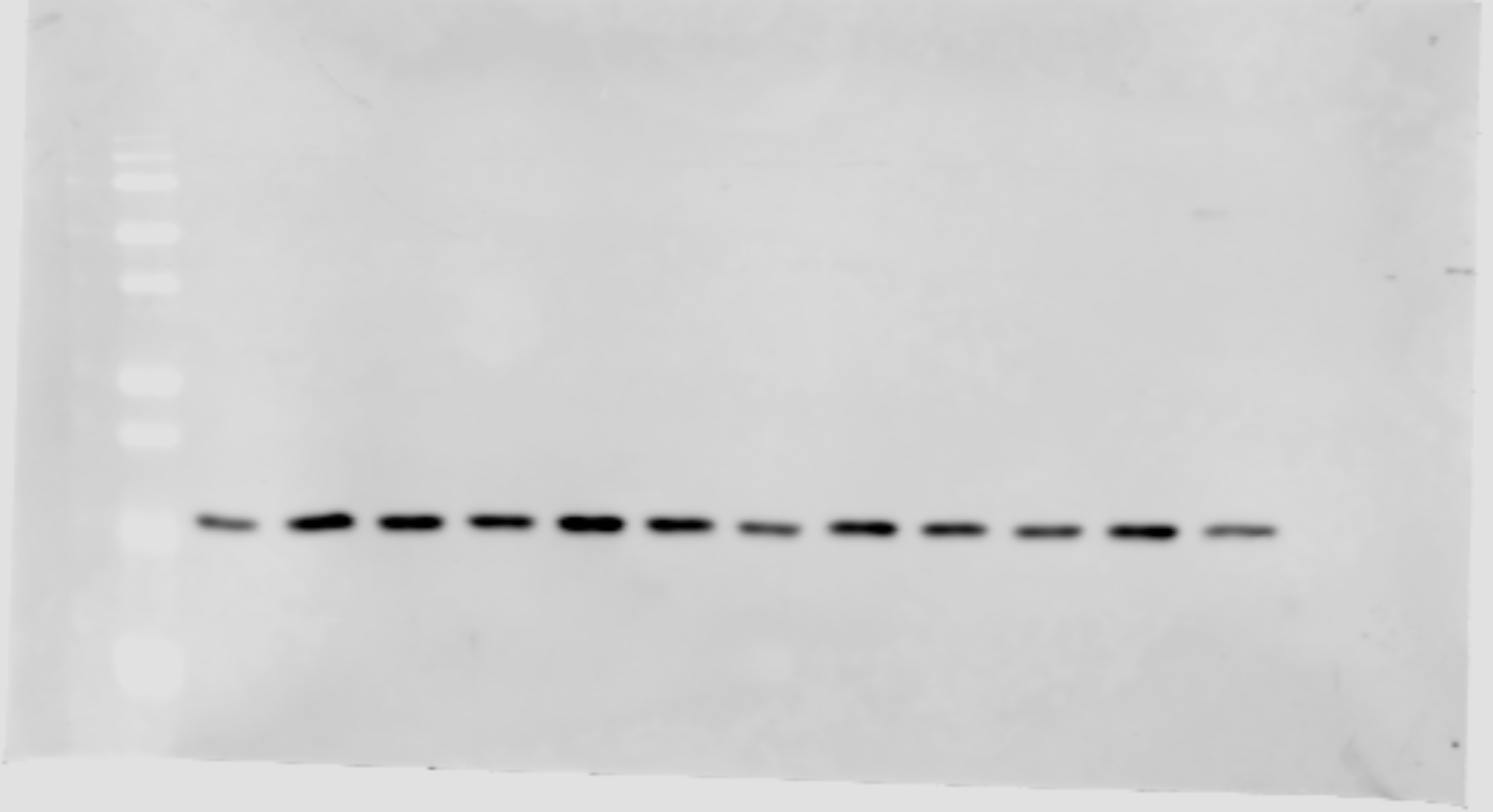

Supplement: Figure 4—figure supplement 1—source data 1. [file elife-60311-fig4-figsupp1-data1.zip › Figure 4-figure supplement 1-source data 1/acH3.png]

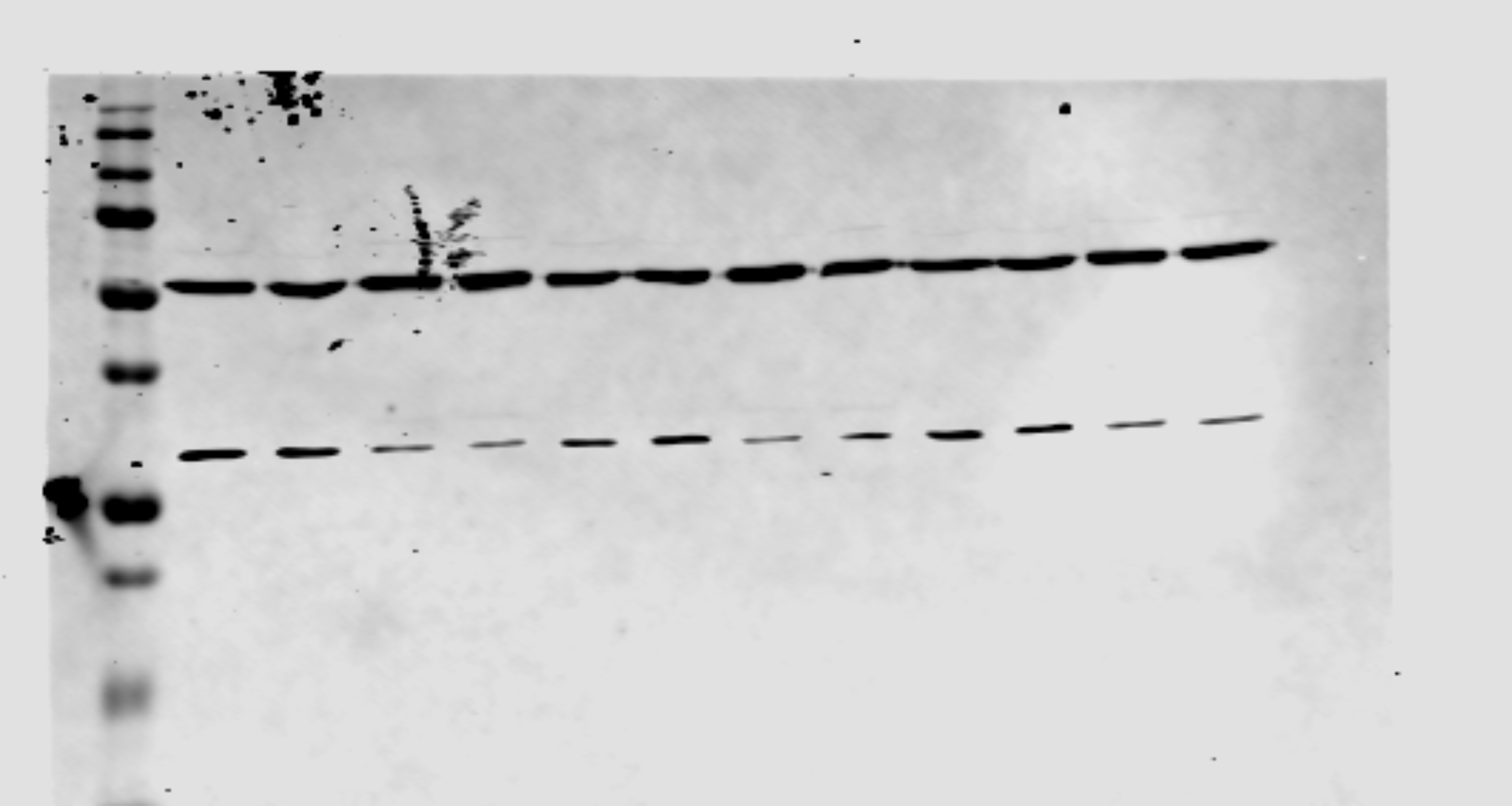

Supplement: Figure 4—figure supplement 1—source data 1. [file elife-60311-fig4-figsupp1-data1.zip › Figure 4-figure supplement 1-source data 1/HO1.png]

Figure 5-source data 2 Original western blots for figure 5

Figure 5 A H3K9ac

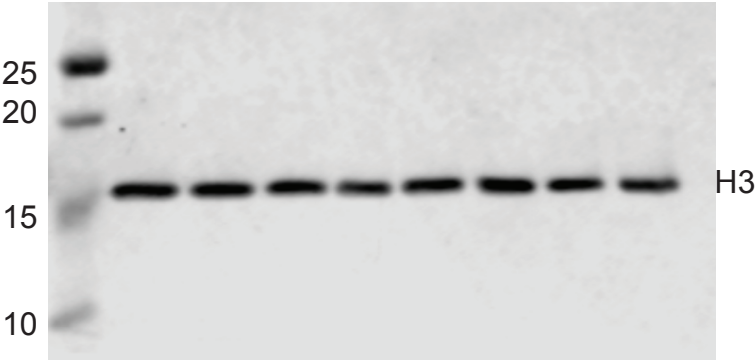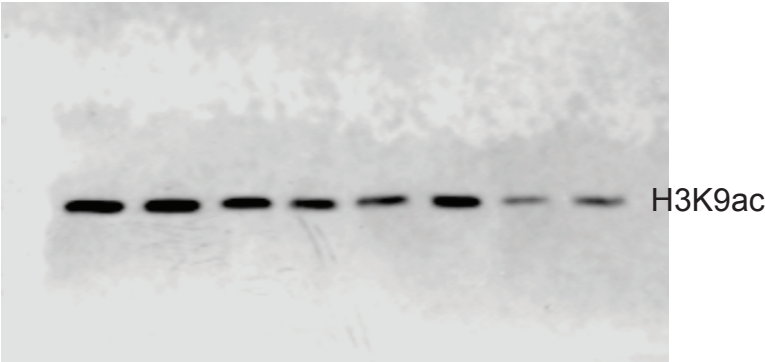

Supplement: Figure 5—source data 2. [file elife-60311-fig5-data2.zip › Figure 5-source data 2/Figure 5-source data 2.pdf]

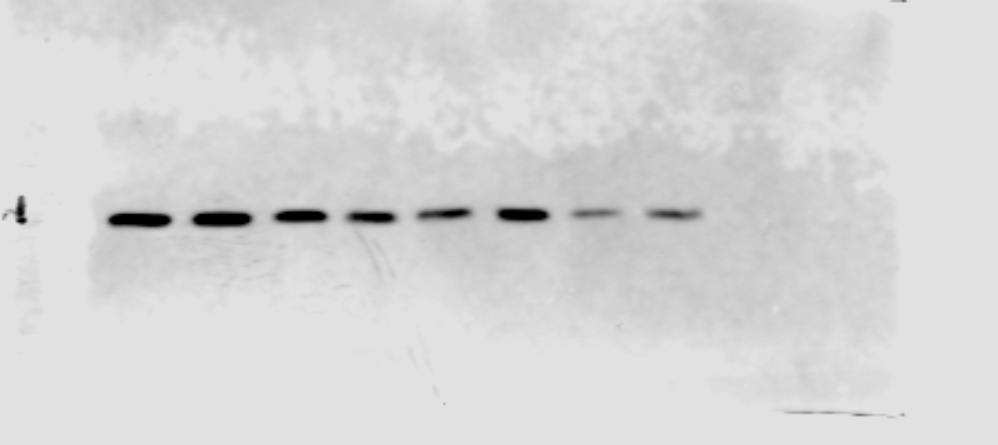

Supplement: Figure 5—source data 2. [file elife-60311-fig5-data2.zip › Figure 5-source data 2/K9ac_MCADKD.png]

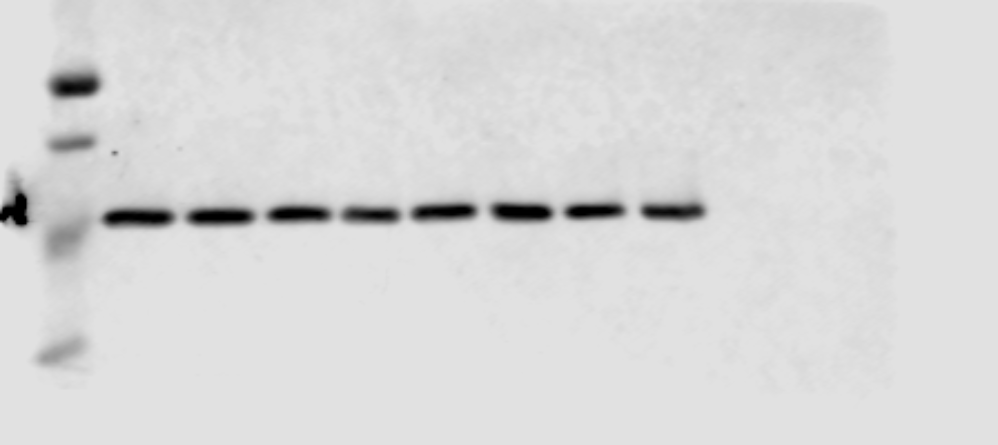

Supplement: Figure 5—source data 2. [file elife-60311-fig5-data2.zip › Figure 5-source data 2/K9ac_MCADKD_H3.png]

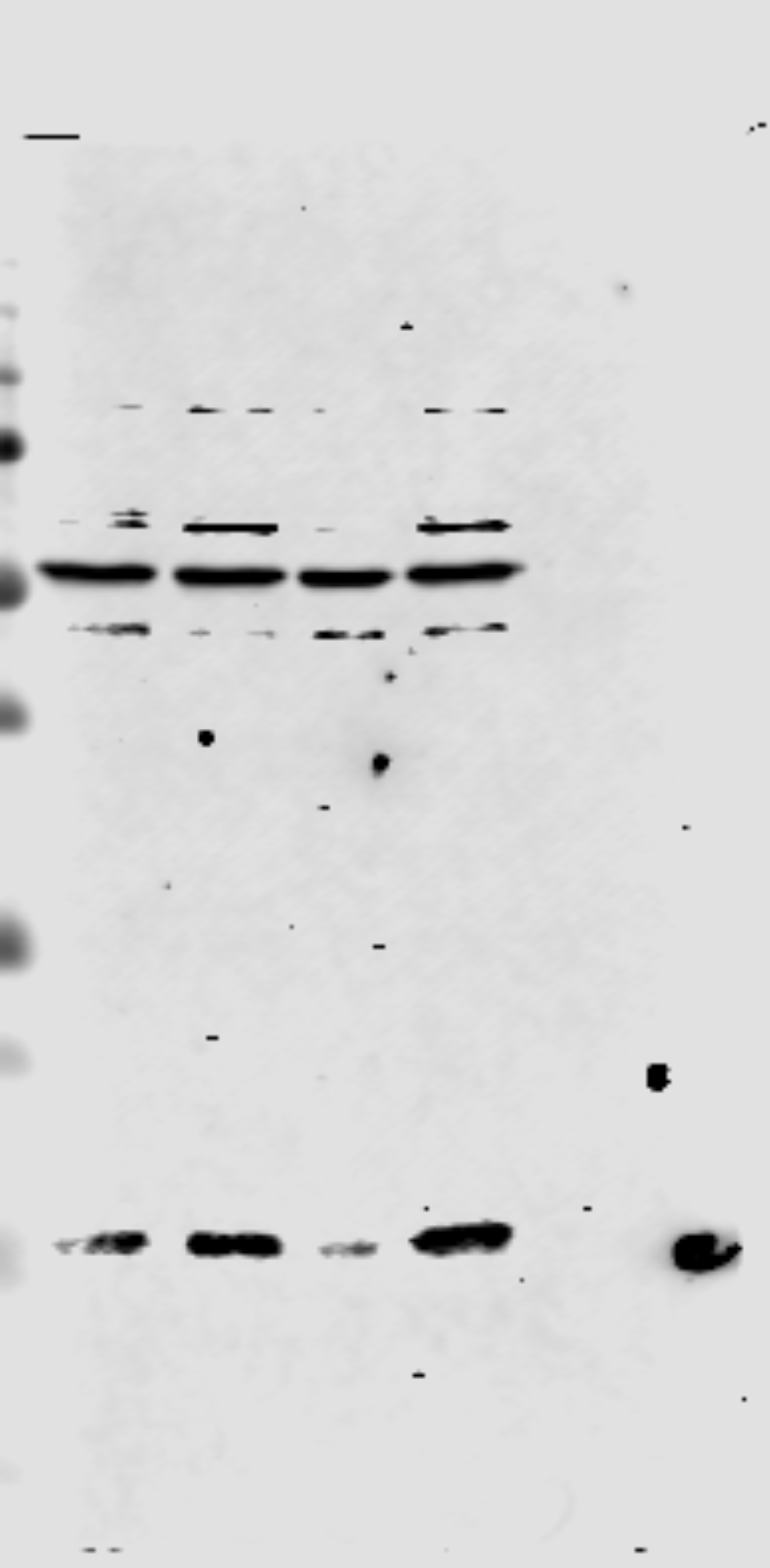

Supplement: Figure 5—figure supplement 1—source data 1. [file elife-60311-fig5-figsupp1-data1.zip › Figure 5-figure supplement 1-source data 1/MCADKD.png]

Figure 5-figure supplement 1- source data 1: Original blots for Figure 5-figure supplement 1

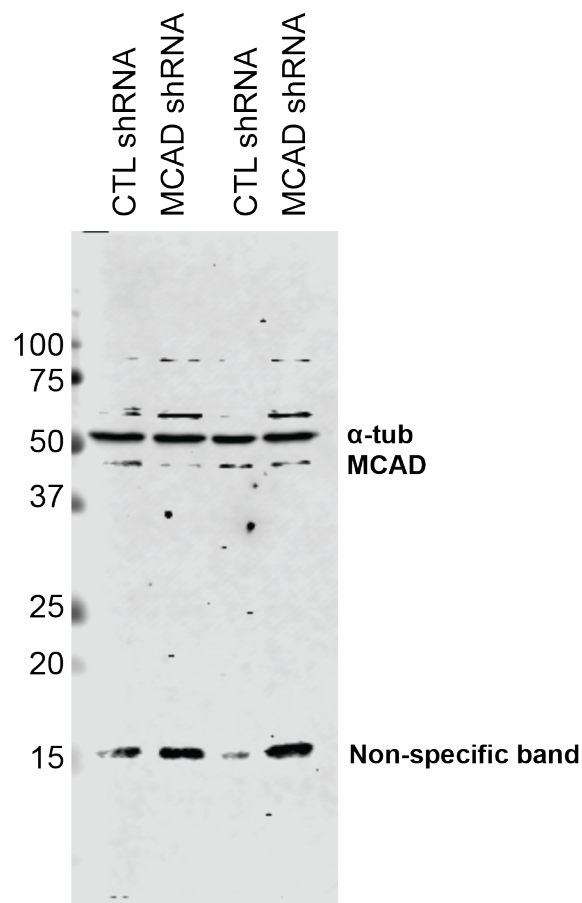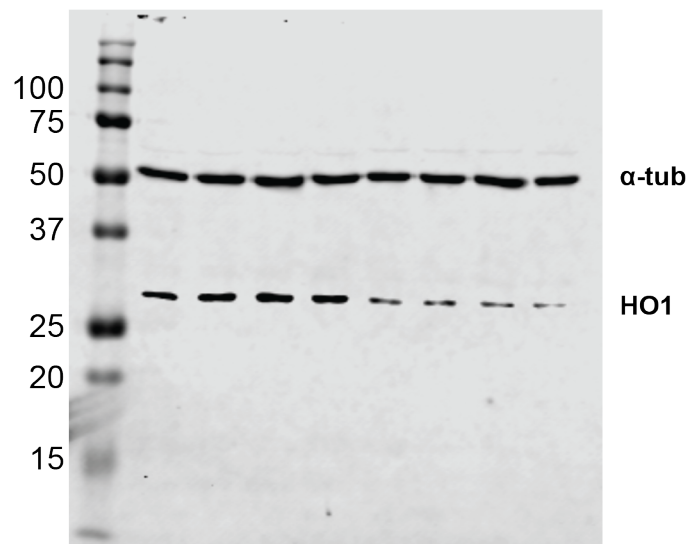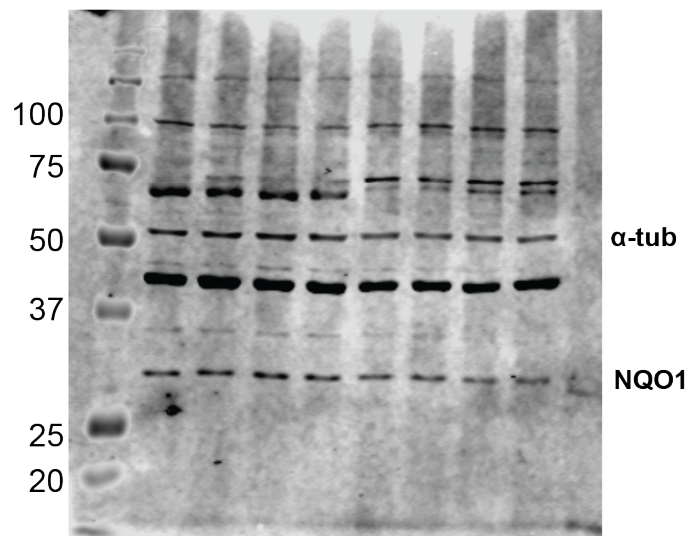

Supplement: Figure 5—figure supplement 1—source data 1. [file elife-60311-fig5-figsupp1-data1.zip › Figure 5-figure supplement 1-source data 1/Figure 5-figure supplement1-source data 1.pdf]

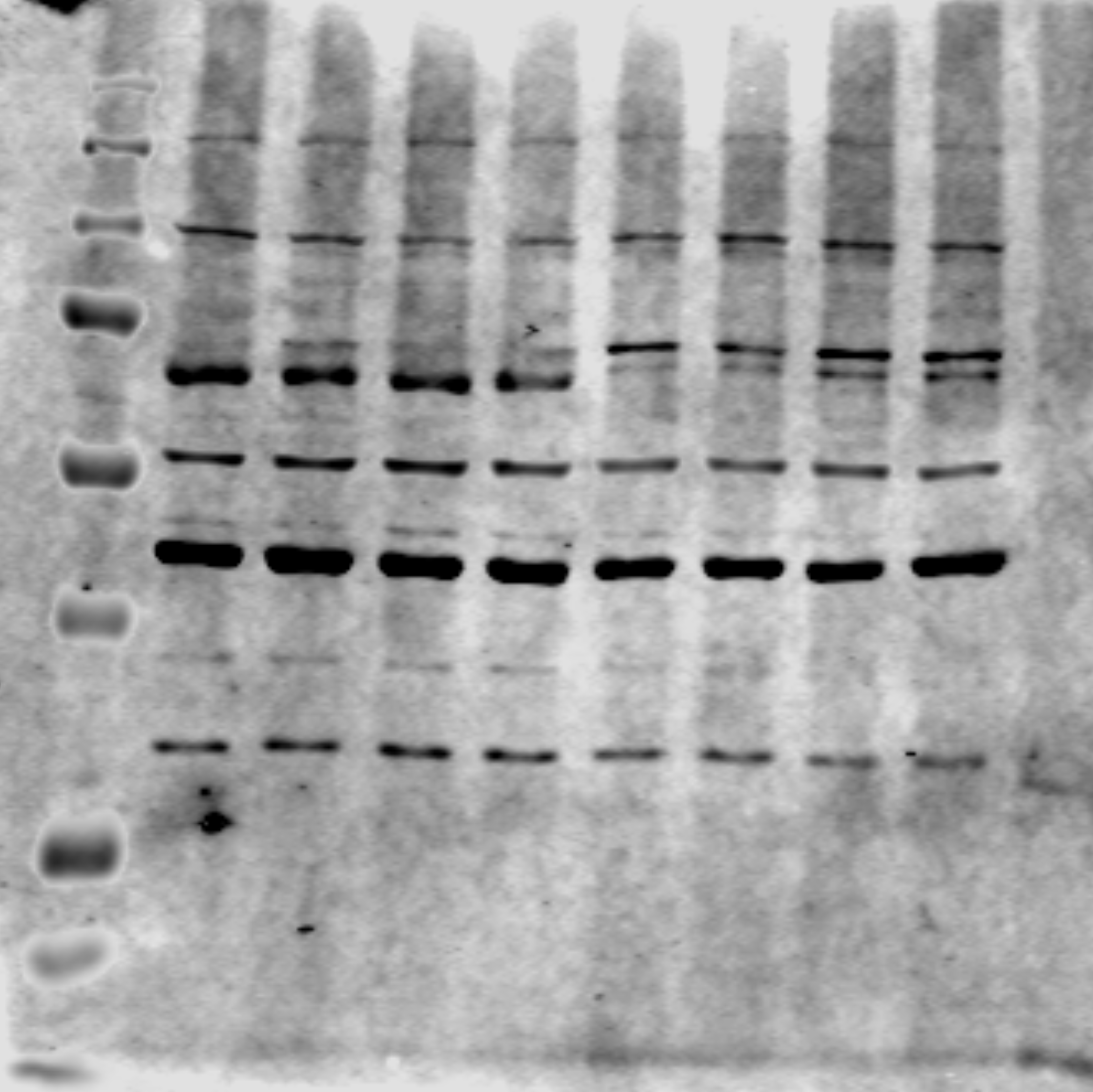

Supplement: Figure 5—figure supplement 1—source data 1. [file elife-60311-fig5-figsupp1-data1.zip › Figure 5-figure supplement 1-source data 1/NQO1.png]

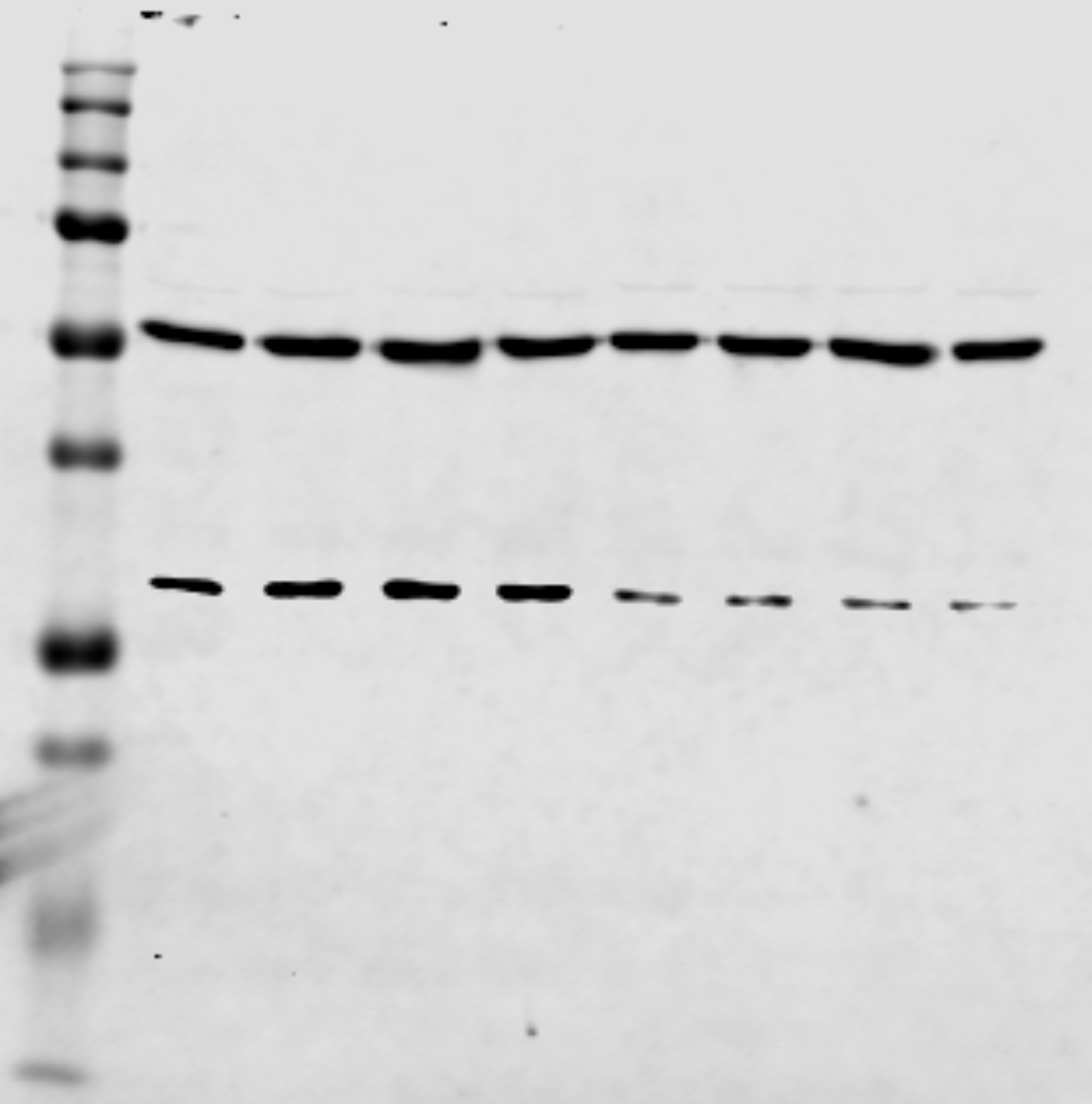

Supplement: Figure 5—figure supplement 1—source data 1. [file elife-60311-fig5-figsupp1-data1.zip › Figure 5-figure supplement 1-source data 1/HO1.png]

Figure 6-source data 2 Original western blots for figure 6

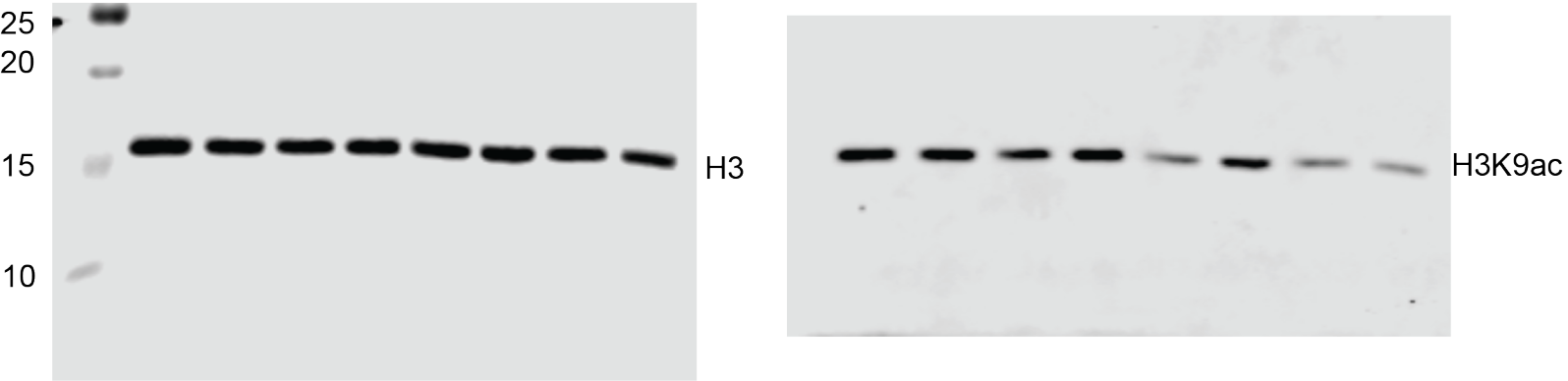

Supplement: Figure 6—source data 2. [file elife-60311-fig6-data2.zip › Figure 6-source data 2/Figure 6-source data 2.pdf]

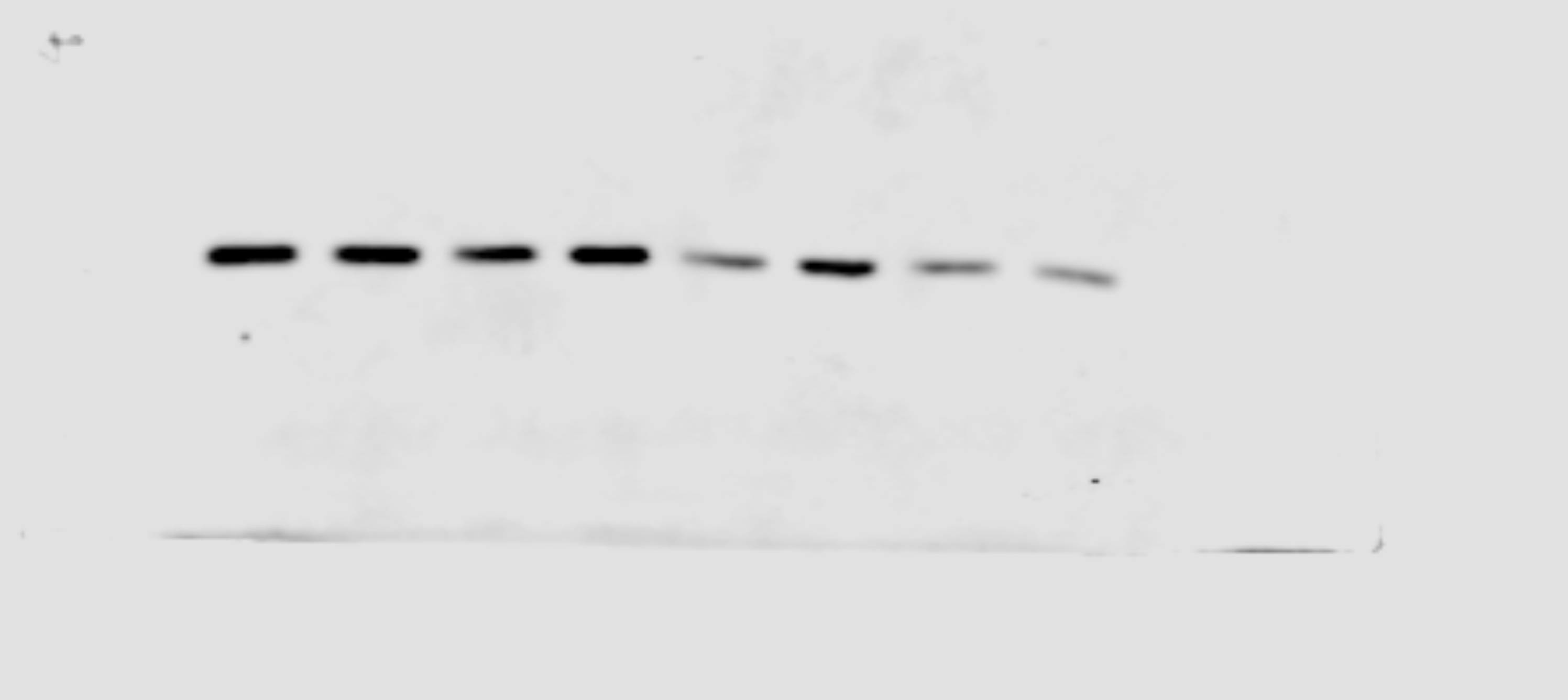

Supplement: Figure 6—source data 2. [file elife-60311-fig6-data2.zip › Figure 6-source data 2/K9ac_Kat2aKD.png]

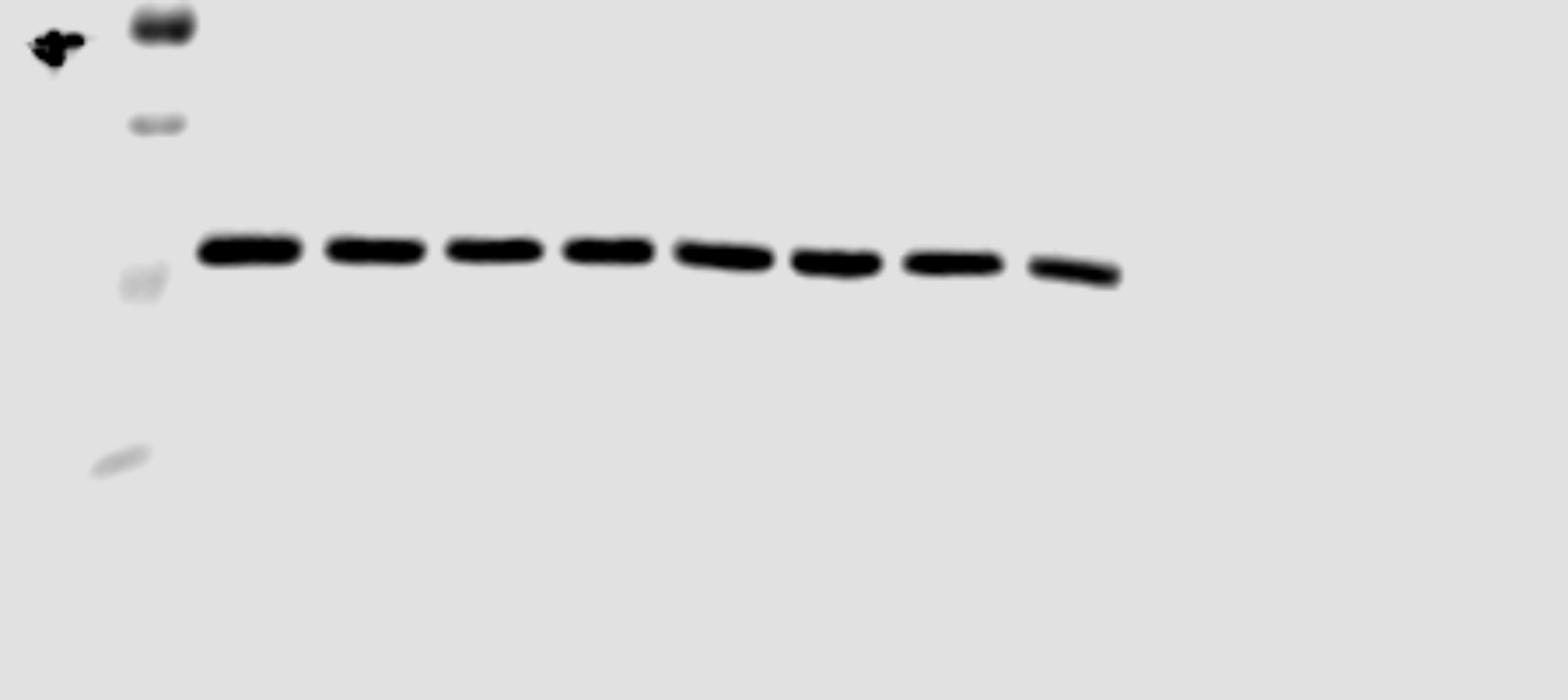

Supplement: Figure 6—source data 2. [file elife-60311-fig6-data2.zip › Figure 6-source data 2/K9ac_H3_Kat2aKD.png]

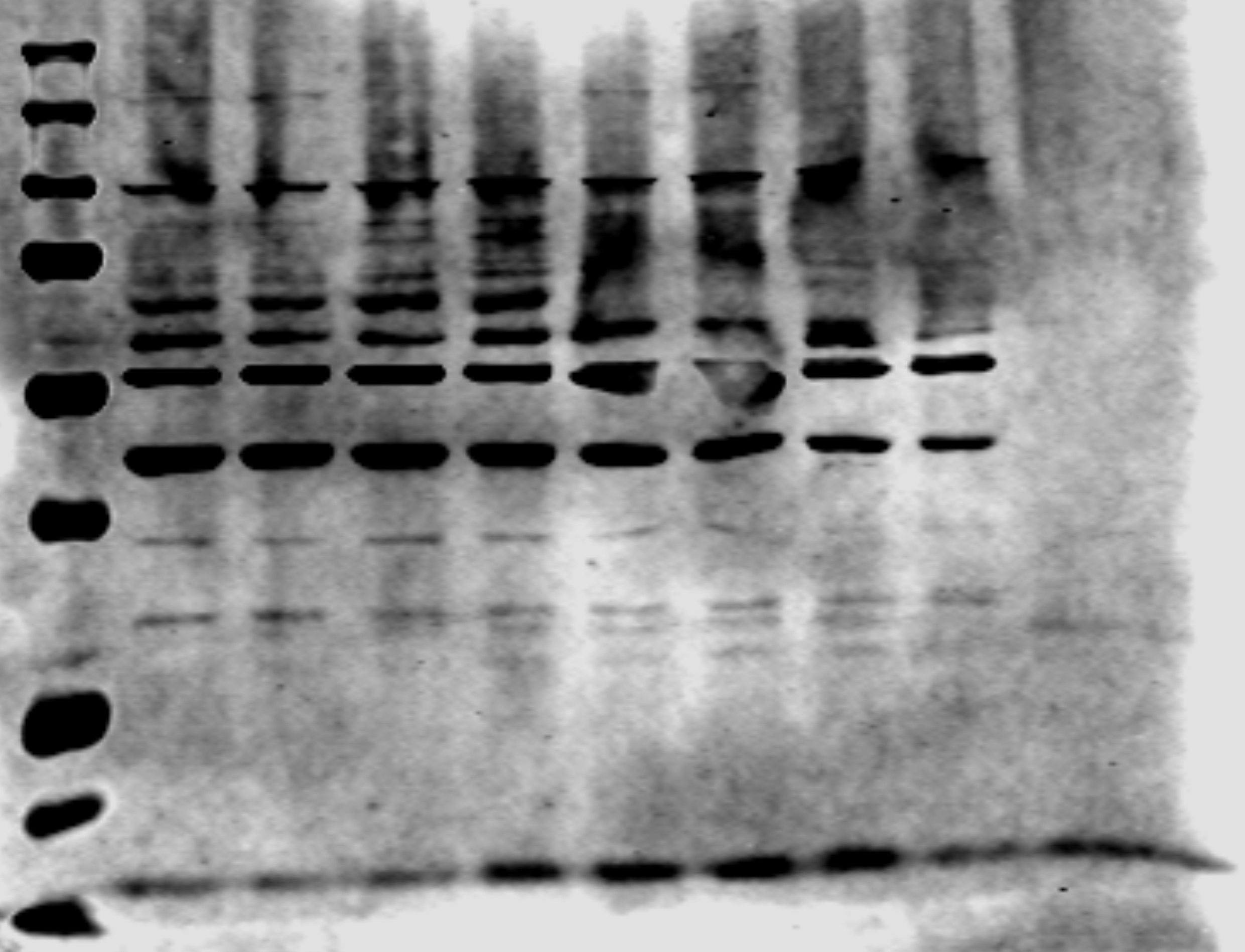

Supplement: Figure 6—figure supplement 1—source data 1. [file elife-60311-fig6-figsupp1-data1.zip › Figure 6-figure supplement 1-source data 1/NQO1.png]

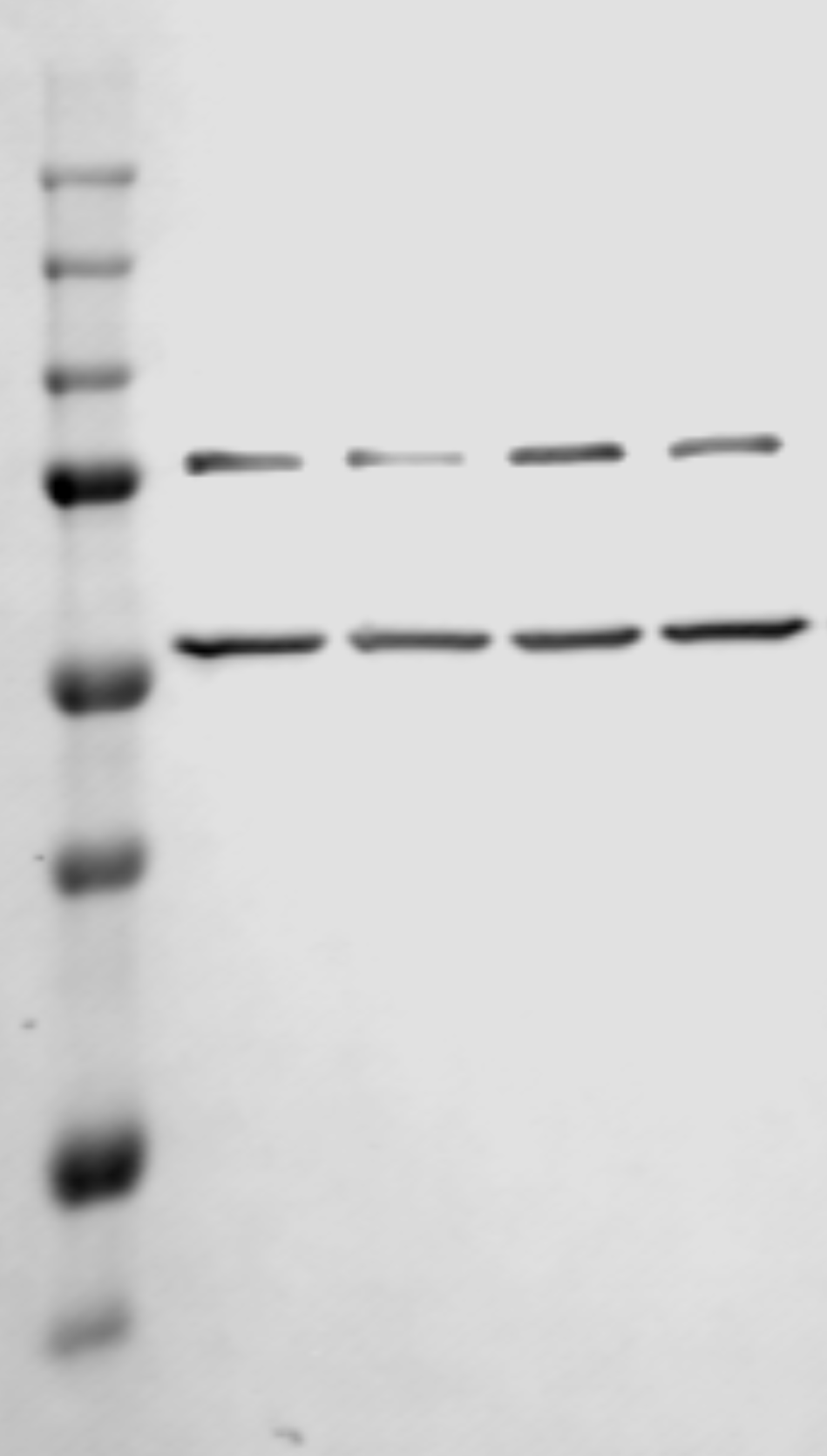

Supplement: Figure 6—figure supplement 1—source data 1. [file elife-60311-fig6-figsupp1-data1.zip › Figure 6-figure supplement 1-source data 1/Kat2a KD.png]

Figure 6-figure supplement 1- source data 1: Original blots for Figure 6-figure supplement 1

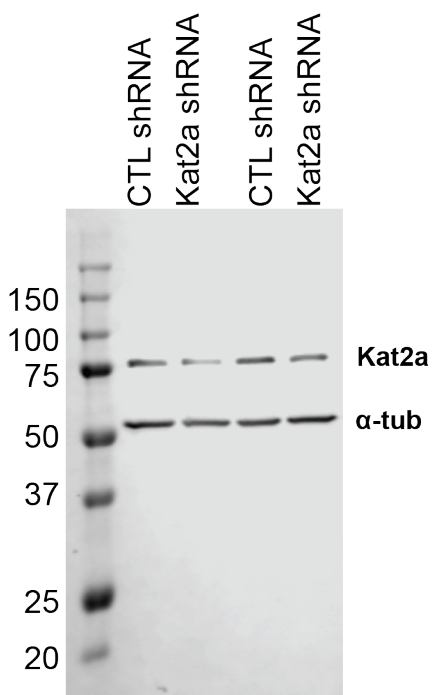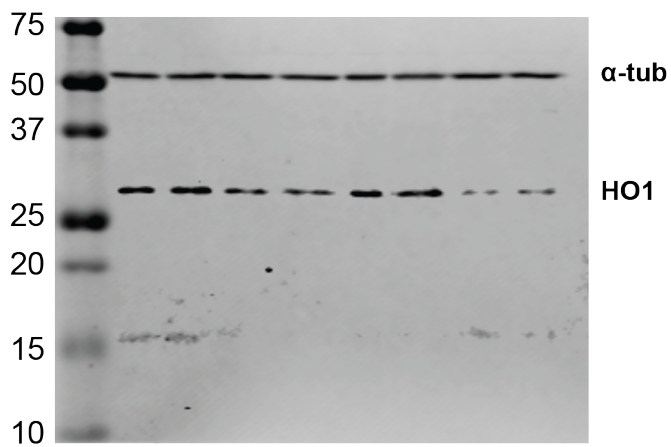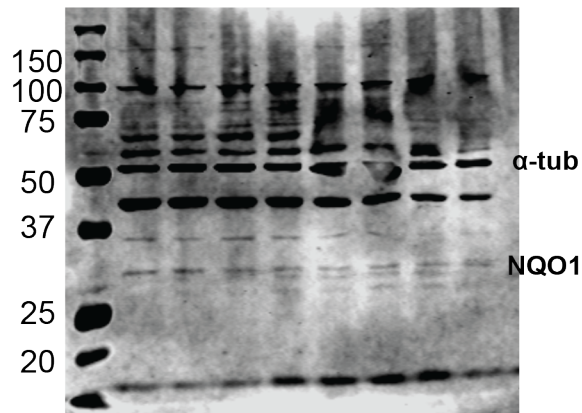

Supplement: Figure 6—figure supplement 1—source data 1. [file elife-60311-fig6-figsupp1-data1.zip › Figure 6-figure supplement 1-source data 1/Figure 6-figure supplement 1-source data 1.pdf]

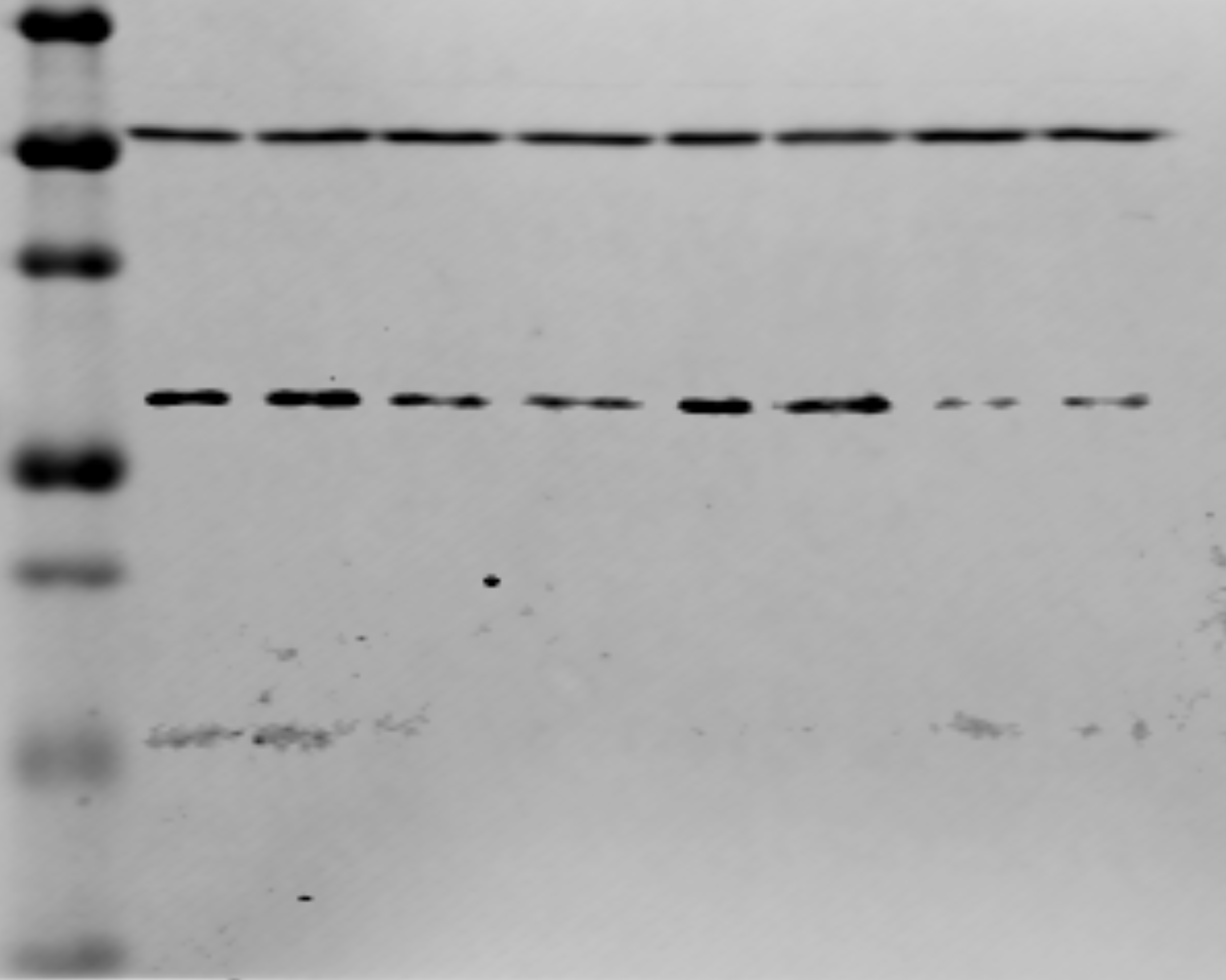

Supplement: Figure 6—figure supplement 1—source data 1. [file elife-60311-fig6-figsupp1-data1.zip › Figure 6-figure supplement 1-source data 1/HO1.png]
